# Is There a Role for Bioactive Lipids in the Pathobiology of Diabetes Mellitus?

**DOI:** 10.3389/fendo.2017.00182

**Published:** 2017-08-02

**Authors:** Undurti N. Das

**Affiliations:** ^1^BioScience Research Centre, Department of Medicine, Gayatri Vidya Parishad Hospital, GVP College of Engineering Campus, Visakhapatnam, India; ^2^UND Life Sciences, Battle Ground, WA, United States

**Keywords:** bioactive lipids, arachidonic acid, lipoxin A4, polyunsaturated fatty acids, resolvins, protectins, maresins

## Abstract

Inflammation, decreased levels of circulating endothelial nitric oxide (eNO) and brain-derived neurotrophic factor (BDNF), altered activity of hypothalamic neurotransmitters (including serotonin and vagal tone) and gut hormones, increased concentrations of free radicals, and imbalance in the levels of bioactive lipids and their pro- and anti-inflammatory metabolites have been suggested to play a role in diabetes mellitus (DM). Type 1 diabetes mellitus (type 1 DM) is due to autoimmune destruction of pancreatic β cells because of enhanced production of IL-6 and tumor necrosis factor-α (TNF-α) and other pro-inflammatory cytokines released by immunocytes infiltrating the pancreas in response to unknown exogenous and endogenous toxin(s). On the other hand, type 2 DM is due to increased peripheral insulin resistance secondary to enhanced production of IL-6 and TNF-α in response to high-fat and/or calorie-rich diet (rich in saturated and trans fats). Type 2 DM is also associated with significant alterations in the production and action of hypothalamic neurotransmitters, eNO, BDNF, free radicals, gut hormones, and vagus nerve activity. Thus, type 1 DM is because of excess production of pro-inflammatory cytokines close to β cells, whereas type 2 DM is due to excess of pro-inflammatory cytokines in the systemic circulation. Hence, methods designed to suppress excess production of pro-inflammatory cytokines may form a new approach to prevent both type 1 and type 2 DM. Roux-en-Y gastric bypass and similar surgeries ameliorate type 2 DM, partly by restoring to normal: gut hormones, hypothalamic neurotransmitters, eNO, vagal activity, gut microbiota, bioactive lipids, BDNF production in the gut and hypothalamus, concentrations of cytokines and free radicals that results in resetting glucose-stimulated insulin production by pancreatic β cells. Our recent studies suggested that bioactive lipids, such as arachidonic acid, eicosapentaneoic acid, and docosahexaenoic acid (which are unsaturated fatty acids) and their anti-inflammatory metabolites: lipoxin A4, resolvins, protectins, and maresins, may have antidiabetic actions. These bioactive lipids have anti-inflammatory actions, enhance eNO, BDNF production, restore hypothalamic dysfunction, enhance vagal tone, modulate production and action of ghrelin, leptin and adiponectin, and influence gut microbiota that may explain their antidiabetic action. These pieces of evidence suggest that methods designed to selectively deliver bioactive lipids to pancreatic β cells, gut, liver, and muscle may prevent type 1 and type 2 DM.

## Introduction

The diabetes mellitus (DM) is classically divided into two types: *type 1 diabetes* that occurs because of autoimmune destruction of β cells that results in insulin insufficiency and so are insulin dependent and *type 2 diabetes* characterized by peripheral insulin resistance and consequent hyperinsulinemia.

## Clinical Manifestations of DM

In majority of the subjects with type 2 DM, no symptoms could be present at the time of detection of the disease. Many a times, type 2 DM is detected during a routine general checkup or when the subject is evaluated for yet another illness. Thus, type 2 DM could be asymptomatic for long periods of time. In an occasional instance, type 2 DM is detected due to the presence of a complication secondary to long-standing diabetes; yet the subject could be unaware of the presence of diabetes. Hence, a high degree of suspicion is necessary on the part of the physician to detect and diagnose type 2 DM. By contrast, type 1 diabetes mellitus (type 1 DM), in general, shows a more dramatic presentation such as diabetic ketoacidosis.

The degree of hyperglycemia may vary depending on the underlying disease process. The underlying metabolic process and factors that modulate it determine the degree of hyperglycemia and, hence, the treatment of diabetes should take into consideration not only the underlying pathobiology but also various factors that have the potential to modify it.

## Classification of DM

Even though diabetes is distinctly divided into different types at the time of diagnosis, many diabetics do not necessarily fit into a single class. For instance, those with gestational diabetes mellitus may have persistent hyperglycemia even after delivery and may be diagnosed to have type 2 DM. By contrast, those who received large doses of corticosteroids may be mistakenly diagnosed to have type 2 DM; yet such an individual may become normoglycemic, once the corticosteroids are withdrawn. Some of those who were given thiazides may develop diabetes after a while. Thiazides by themselves are not diabetogenic; such individuals probably had type 2 DM that is precipitated by thiazides. Thus, it is less important to label a diabetes but it is important to understand the significance and consequences of and to treat it as effectively as possible.

## Type 1 DM

Type 1 diabetes that occurs in 5–10% of those with diabetes is due to insulin insufficiency due to destruction of β cells. Those who are at risk of type 1 diabetes generally show islet cell antibodies, anti-insulin antibodies, antibodies against glutamic acid decarboxylase (GAD_65_), and antibodies against tyrosine phosphatases 1A-2 and 1A-2β. About ~85–90% of these patients have more than one of these autoantibodies. A close relationship exists between HLA markers: HLA-DQA and DQB genes and type 1 DM, which (DR/DQ alleles) can be either predisposing or protective. In general, subjects with type 1 DM may also have other autoimmune diseases (AID).

## Pathobiology of Type 1 Diabetes

In general, it is believed that excess production of interleukin-1 (IL-1), IL-2, IL-6, tumor necrosis factor-α (TNF-α) and macrophage migration inhibitory factor (MIF), nitric oxide (NO), superoxide anion, and other related or similar free radicals plays a significant role in the pathobiology of type 1 DM. Macrophages, lymphocytes, and monocytes infiltrate pancreatic β cells and release cytotoxic molecules leading to the development of type 1 DM (1, 2). Streptozotocin (STZ) induces significant production of IL-2, interferon-γ (IFN-γ), and TNF-α by T_H_1 lymphocytes, which activates macrophages, leading to the production of excess of nitric oxide (NO) and other nitroso compounds to induce apoptosis of β cells (3) Human duct cells situated close to β cells produce TNF-α, which can induce death of pancreatic β cells (4). Macrophages produce MIF that plays a significant role in type 1 DM (5). Non-obese diabetic (NOD) mice when administered recombinant MIF-protein two times a week (from age 6 to 11 weeks) show enhanced incidence of type 1 DM compared with untreated control (5). TNF-α upregulates MIF production (6, 7) and both TNF-α and MIF act in synergy to induce type 1 DM.

Migration inhibitory factor, TNF-α, and ILs enhance the synthesis and release of pro-inflammatory prostaglandins (PGs) by increasing the expression of COX-2 mRNA but, paradoxically, suppress prostacyclin synthase (PGI_2_S) mRNA expression, leading to decreased PGI_2_ production. By contrast, at low concentrations TNF-α decreased, whereas IL-1β enhanced PGI_2_ production in a dose-dependent manner. Paradoxically, low amounts of TNF-α and MIF enhanced PGI_2_ synthesis, but to a much lesser degree. Thus, an interaction exists between cytokines and PGs (8–10).

Furthermore, arachidonic acid (AA)-derived prostaglandin E_2_ (PGE_2_) inhibits TNF-α and IL-1 production (11) implying that TNF-α, IL-1-induced enhancement of PGE_2_ has a negative regulatory control on these cytokines and, thus, modulate the actions of pro-inflammatory cytokines on the induction of type 1 DM. For instance, peroxisome proliferator-activated receptor-γ (PPAR-γ) activators: conjugated linoleic acid (CLA) and troglitazone (12) inhibit free radical generation and TNF-α and IL-2 and, thus, inhibit the occurrence of diabetes in the Zucker diabetic fatty fa/fa rat (13, 14).

Previously, we reported that oral supplementation of polyunsaturated fatty acid (PUFAs)-rich oils and pure individual PUFAs, such as γ-linolenic acid (GLA, 18:3 n-6), arachidonic acid (AA, 20:4 n-6), eicosapentaenoic acid (EPA, 20:5 n-3), and docosahexaenoic acid (DHA, 22:6 n-3) that serve as endogenous ligands of PPARs (12) prevented development of alloxan-induced DM in experimental animals (15–18). Free radical-induced DNA damage activates poly (ADP-ribose) polymerase (PARP) synthase (19) resulting in enhanced NAD^+^ utilization and because of which NAD^+^ depletion occurs. This leads to a significant decrease or complete depletion of NAD^+^-dependent energy generation that results in alterations in protein metabolism resulting in pancreatic β cell death (19, 20). The fact that nicotinamide supplementation suppresses free radical generation and, thus, ameliorates DM is in support of the role of PARP and free radicals in the pathogenesis of DM. Thus, the protective actin of PUFAs we observed (15–18) could be due to their ability to prevent apoptosis of pancreatic β cells by restoring to normal altered lipid peroxides, NO, superoxide dismutase (SOD), ceruloplasmin, glutathione peroxidase, glutathione-S-transferase, and catalase. NO quenches superoxide anion (21, 22), whereas SOD inactivates superoxide anion. In our study (17, 18), we noted that PUFAs restored SOD and NO to normal, which is one mechanism by which they prevent alloxan-induced type 1 DM.

Recently, we noted that even STZ-induced type 1 and type 2 DM and high-fat diet (rich in saturated fats and high amounts of trans fats) induced type 2 DM can also be prevented by both oral and intraperitoneal administration of AA, suggesting that AA has potent cytoprotective action both *in vitro* and *in vivo* (unpublished data). In an extension of this work, it was observed that the pancreatic β cell protective action of AA is not blocked by both cyclo-oxygenase (COX) and lipoxygenase (LOX) inhibitors implying that there is no significant role for PGs, leukotrienes (LTs), and thromboxanes (TXs) in the cytoprotective action offered by AA [Ref. (17, 18) and see below].

In an extension of these studies, it was noted that lipoxin A4 (LXA4), an anti-inflammatory metabolite of AA, not only protected pancreatic β cells from the cytotoxic actions of alloxan and STZ *in vitro* but also prevented both alloxan-induced type 1 DM and STZ-induced type 1 and type 2 DM in experimental animals (see below). LXA4 restored to normal altered antioxidant concentrations, and expressions of Pdx1, NF-kB, and IKB genes in the pancreas and plasma TNF-α levels in type 1 and type 2 DM; Nrf2, Glut2; COX-2 and inducible nitric oxide (iNOS) proteins in pancreatic tissue of type 1 DM and LPCLN2 (lipocalin 2), NF-kB, IKB I in adipose tissue of type 2 DM to normal. PDX1 is a homeobox protein expressed in β pancreatic cells that maintains and expresses the endocrine function of the pancreas. These results imply that there are some endogenous anti-inflammatory molecules that can protect β cells and prevent both type 1 and type 2 DM. Based on our studies, we suggest that AA and LXA4 belong to this category.

It is interesting to note that other unsaturated fatty acids: gamma-linolenic acid (GLA, 18:3 n-6), eicosapentaenoic acid (EPA, 20:5 n-3), and docosahexaenoic acid (DHA, 22: n3) also showed cytoprotective action against alloxan and STZ-induced toxicity to pancreatic β cells *in vitro* and development of alloxan-induced type 1 and STZ-induced type 1 and type 2 DM in experimental animals, though their beneficial actions were much less potent compared to AA (15–18). What is interesting is the observation that GLA, EPA, and DHA also enhanced the formation of LXA4 though much less potent compared to AA. It is possible that, especially EPA and DHA displace AA from the cell membrane lipid pool and, thus, enhance the production of LXA4. This implies that even GLA, EPA, and DHA may bring about their beneficial action by enhancing the production of LXA4.

In an extension of these studies, we also noted that anti-inflammatory metabolites of EPA and DHA, such as resolvins and protectins, are ineffective in preventing alloxan and STZ-induced cytotoxicity against pancreatic β cells *in vitro* but, paradoxically, prevented development of STZ-induced type 2 DM in experimental animals (see below). The exact reason for this discrepancy between *in vitro* and *in vivo* results is not clear. One possibility is that the anti-inflammatory actions of resolvins and protectins can suppress the peripheral insulin resistance seen in type 2 DM and/or able to trigger the production of other antidiabetic molecules, such as brain-derived neurotrophic factor (BDNF). Though it is not yet certain but our studies showed that adipose tissue and liver are the primary targets of resolvins and protectins unlike LXA4 that targets specifically pancreatic β cells and possibly, LXA4 is a more potent anti-inflammatory molecule. Thus, it is likely that although LXA4, resolvins, protectins, and maresins are all anti-inflammatory molecules, their targets are different and so a difference in their antidiabetic actions.

Yet another molecule that may have a role in type 1 DM is nitric oxide (NO). Supraphysiological amounts of nitric oxide (NO) produced by induction of iNOS (inducible nitric oxide synthase) are toxic to pancreatic β cells. Both macrophage and β cell produced NO-induced β cell lysis (23, 24) by damaging DNA (25). This leads to activation of ADP-ribose polymerase in islet cells (26) resulting in a significant decrease in intracellular NAD^+^ and as a result, insufficient energy generation occurs leading to β cell apoptosis. This is supported by the observation that mice lacking PARP (poly-ADP-ribose polymerase) gene are resistant to diabetes induced by STZ (27). It is possible, but needs to be documented, that PUFAs block PARP and, thus, bring about their antidiabetic action against alloxan and STZ-induced type 1 DM. Oral administration of cod liver oil, a good source of ω-3 EPA and DHA, during pregnancy decreased the incidence of type 1 DM (28) that is in support of our animal studies in which we observed that PUFAs prevented type 1 DM (16–18). Based on these studies (16–28), it is likely that lower intake of PUFAs during pregnancy and lactation by the mother and during perinatal period by the newborn may contribute to the development of type 1 DM.

## Autoimmune Type 1 DM

IL-1β, TNF-α, and IFN-γ produced by islet-infiltrating T cells and macrophages induce apoptosis or dysfunction of pancreatic β cells by enhancing the formation of oxygen free radicals, nitric oxide, and peroxynitrite (29, 30). Studies revealed that enteroviruses accelerate the development of type 1 DM, in part, due to their tropism for β cells and ability to replicate in β cells at an appropriate and precisely right time to induce the diabetogenic process. In addition, expression of class-I major histocompatibility complexes, toll-like receptor-dependent immunity, and interferon pathways have a significant role in the development of diabetes. By contrast, type 1 DM can be prevented by blocking anti-viral responses, inhibition of autoreactive memory effector T cells, and enhancement of regulatory T cell (Treg) function. In this context, induction of immunoregulatory mechanisms, especially the function of Tregs, is of therapeutic interest (31).

Recent studies suggested that intestinal flora have a significant role in the pathobiology of type 1 DM. It was reported that bacteria entering the pancreatic ductal system can trigger β-cell destruction in experimental animals. Preliminary evidence did suggest that such an event could also occur in humans with type 1 DM. Instillation of bacterial species that are normally present in the human duodenum into the healthy rat pancreatic ductal system induced infiltration of neutrophil polymorphonuclear cells and monocytes/macrophages around the pancreatic ducts, which released IL-6, IL-8, and monocyte chemotactic protein 1 that, in turn, produced hydropic degeneration of β cells, an event that is very much similar with the morphologic findings seen in patients dying with type 1 DM. These results strongly suggest that bacteria can elicit an adverse innate immunity response (32).

These observations are supported by the reports that normal controls have microbiota that produce higher amounts of butyrate and lactate that aids to induce production of enough mucin synthesis that maintains gut integrity. On the other hand, non-butyrate-producing lactate-utilizing bacteria are present in higher amounts in subjects with type 1 DM because of which they fail to produce optimum amounts of mucin (33).

Type 1 diabetes mellitus does not occur in pathogen-free NOD mice lacking MyD88 protein (an adaptor for multiple innate immune receptors that recognize microbial stimuli), and this has been attributed to the presence of commensal microbes. This is so since, germ-free MyD88-negative NOD mice develop diabetes, whereas colonization of these germ-free MyD88-negative NOD mice with healthy gut bacterial phyla do not develop type 1 DM. This implies that MyD88 deficiency can change the gut microbiota composition, and exposure to specific microbiota can influence their susceptibility or resistance to the development of type 1 DM. These results confirm the interaction between intestinal microbes with the innate immune system that seems to have a critical epigenetic role in the development of type 1 DM (34). For instance, abundance of *Bacteroides* and deficiency of butyrate-producing bacteria in gut is associated with β cell autoimmunity and type 1 DM, suggesting that altered gut microbiota results in immunological aberrations that paves way for the development of the disease. It is likely that changes in gut microbiota alters the gut immune system such that there could be an increase in gut permeability, enhanced small intestinal inflammation, and impaired tolerance to food antigens, events seen in type 1 DM. This alteration in gut microbiota may explain why type 1 DM patients are more prone to enterovirus infections, and do not develop tolerance to cow milk antigens. This complex interaction among gut microbiota, host, environment, and disease mechanisms need further studies to develop novel targets in the prevention of type 1 DM (35).

## Immunotherapy of Type 1 DM

The involvement of immune mechanisms in the pathobiology of type 1 DM is supported by the reports that Bio-Breeding (BB) rats do not develop hyperglycemia when treated with anti-lymphocyte serum, by transfusion of normal T cells, and cyclosporin A (36–38). The NOD mouse is an animal model of type I DM that shows insulitis, infiltration of macrophages and lymphocytes into the islets, and reduction of islet size and a perturbed immune system (39). These NOD mice have impaired cell-mediated immunity, including an absolute decrease in T-cell activity, production of IL-2 by spleen cells and proliferation of the spleen cells induced by IL-2 are very low (39). These results are supported by the observation that NOD mice are protected from the development of insulitis and diabetes by strategies designed to activate macrophages and killer T-cells, enhancing interferon production and increasing IL-2 synthesis (40–43).

For example, OK-432 (a streptococcal preparation), a potent activator of both macrophages and killer T cells and an enhancer of IL-2 production, inhibited development of diabetes in all the treated animals over 24-week observation period compared to control (39). OK-432-treated NOD mice showed a significant increase both in the number of the mononuclear spleen cells and their natural killer cell activity and had few effector cells that induce apoptosis of pancreatic β cells (44).

**Figure 1:**
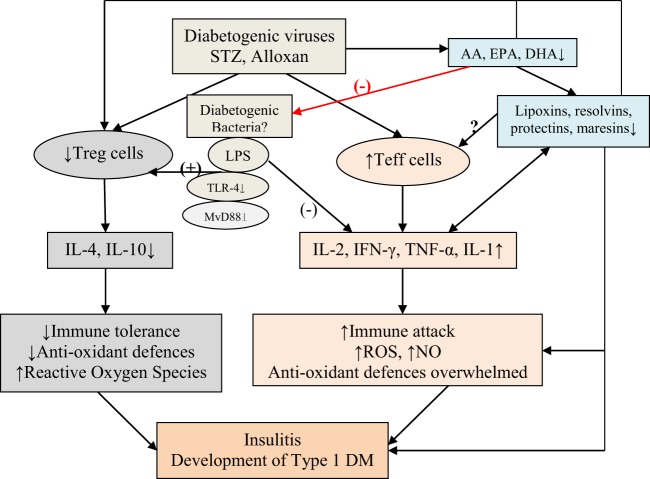
Scheme showing probable mechanism by which diabetogenic viruses, streptozotocin (STZ), and alloxan induce the development of type 1 diabetes mellitus (type 1 DM). The same mechanism may occur in non-obese diabetic (NOD) and other animals that are known to develop type 1 DM. Bacterial endotoxin lipopolysaccharide (LPS), an agonist of toll-like receptor-4 (TLR-4), inhibits type 1 DM. LPS administered to NOD mice during the prediabetic state delays the onset and decreases the incidence of diabetes. A multiple-injection protocol of LPS is more effective than a single LPS intervention. LPS administration suppresses spleen T lymphocyte proliferation, increases the generation of T regulatory cells [indicated as (+) in the figure], and reduces the synthesis of T-helper 1 pro-inflammatory cytokines [indicated as (−) in the figure], and downregulates TLR-4 and its downstream MyD88-dependent signaling pathway and enhances IL-4 and IL-10. Multiple injections of LPS induce tolerogenic dendritic cell (DC) subset with low TLR-4 expression and, thus, prevent development of type 1 DM in NOD diabetic mice see text, Figure 2, and Wang et al. (47). Alloxan and STZ and other diabetogenic molecules, including viruses, may block activity of desaturases and, thus, decrease the formation of arachidonic acid, eicosapentaneoic acid, and docosahexaenoic acid that, in turn, leads to deficiency of lipoxins, resolvins, protectins, and maresins, potent anti-inflammatory substances. Polyunsaturated fatty acid and their products may alter gut microbiota and regulate Treg and Teff cells. Bioactive lipids inhibit production of pro-inflammatory cytokines and possess cytoprotective actions that may explain their ability to prevent type 1 DM (see Figures 6–8).

**Figure 2:**
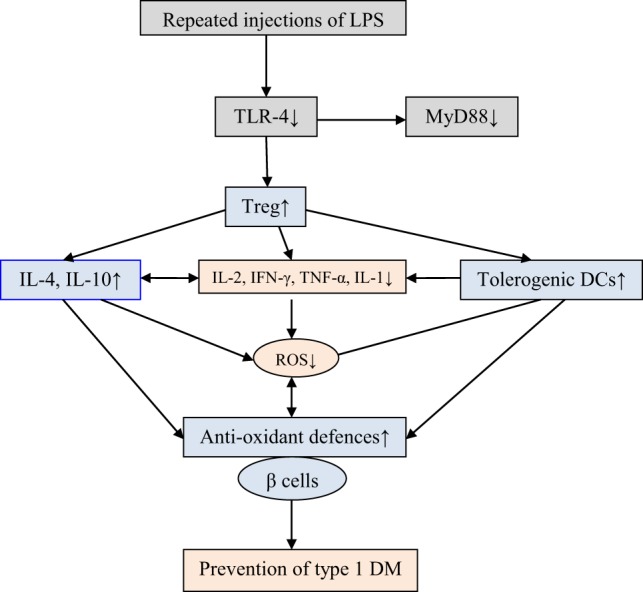
Multiple-injections of lipopolysaccharide (LPS) is effective in preventing type 1 diabetes mellitus (type 1 DM). LPS administration suppresses spleen T lymphocyte proliferation, increases the generation of T regulatory, reduces the synthesis of T-helper 1 pro-inflammatory cytokines [interleukin-2 (IL-2), interleukin-1 (IL-1), interferon-γ (IFN-γ), and tumor necrosis factor-α (TNF-α)], and downregulates toll-like receptor-4 (TLR-4) and its downstream MyD88-dependent signaling pathway and enhances IL-4 and IL-10 and antioxidant defenses. Multiple injections of LPS induce tolerogenic dendritic cell (DC) subset with low TLR-4 expression and, thus, prevent development of type 1 DM in non-obese diabetic mice [see text and Wang et al. (47)].

**Figure 3:**
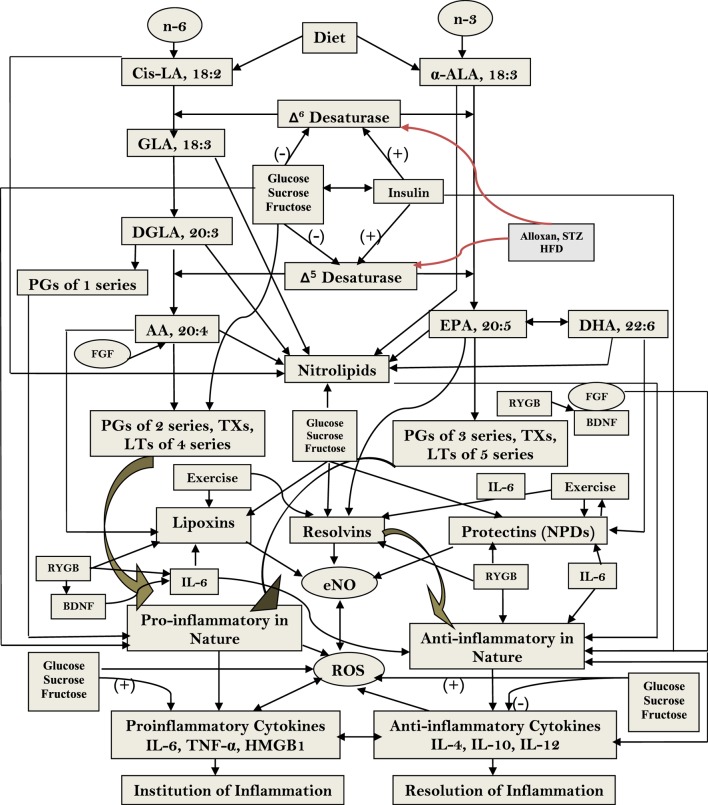
Scheme showing the metabolism of essential fatty acids, their role in inflammation and the effect of glucose, sucrose, and fructose on the activities of desaturases and formation of various eicosanoids, cytokines, and lipoxins (LXs), resolvins, and protectins. (+) Indicates increase in the activity or enhanced formation. (−) Indicates decrease in the activity or decreased formation. Glucose, sucrose, and fructose decrease activities of Δ^6^ and Δ^5^ desaturases and, thus, decrease the formation of arachidonic acid (AA), eicosapentaneoic acid (EPA), and docosahexaenoic acid (DHA) that are precursors of various eicosanoids and LXs, resolvins, and protectins. Glucose, sucrose, and fructose seem to enhance the formation of pro-inflammatory prostaglandins, leukotrienes, and thromboxanes and generation of free radicals and decrease the formation of LXs, resolvins, and protectins that have anti-inflammatory activities and prevent development of type 2 diabetes mellitus and metabolic syndrome and insulin resistance; they may also enhance the formation of pro-inflammatory cytokines and decrease those of anti-inflammatory cytokines. The pro-inflammatory activities of glucose, fructose, and sucrose may be in the order of fructose > sucrose ≥ glucose. Nitrolipids are formed due to interaction between polyunsaturated fatty acids and nitric oxide and these compounds have anti-inflammatory activity. Fibroblast growth factor 1 (FGF1) is a critical transducer of remodeling of adipose tissue in response to fluctuations in nutrient availability that is essential for maintaining metabolic homeostasis and is regulated by the nuclear receptor peroxisome proliferator-activated receptor-γ (PPAR-γ). PPAR-γ is an adipocyte master regulator and the target of the thiazolidinedione class of insulin-sensitizing drugs. FGF1 is the prototype of the 22-member FGF family of proteins and is involved in a range of physiological processes, including development, wound healing, and cardiovascular changes. FGF1 is highly induced in adipose tissue in response to a high-fat diet and that mice lacking FGF1 develop an aggressive diabetic phenotype coupled to aberrant adipose expansion when challenged with a high-fat diet. FGF1-deficient mice have abnormalities in the vasculature network, an accentuated inflammatory response, aberrant adipocyte size distribution, and ectopic expression of pancreatic lipases. It is interesting that withdrawal of the high-fat diet, inflamed adipose tissue fails to properly resolve, resulting in extensive fat necrosis that could be attributed to decreased production of LXs, resolvins, protectins, and maresins. Adipose induction of FGF1 in the fed state is regulated by PPAR-γ acting through a conserved promoter proximal PPAR response element within the FGF1 gene. These results suggest that the PPAR-γ–FGF1 axis is critical for maintaining metabolic homeostasis and insulin sensitization (48). In this context, FGF-19 has been shown to have hypoglycemic actions. Central nervous system responds to FGF-19 administered in the periphery. In mouse models of insulin resistance, leptin-deficiency and high-fat diet feeding and intracerebroventricular infusions of FGF-19 improved glycemic status, reduced insulin resistance and potentiated insulin signaling in the periphery. In addition, central action of FGF-19 included suppression of AGRP/neuropeptide Y neuronal activity (49). Furthermore, high-fat diet (HFD)-fed mice lacking lysosome-associated membrane protein-2 (lamp-2), which is essential for the fusion with lysosome and subsequent degradation of autophagosomes, showed a resistance against HFD-induced obesity, hyperinsulinemic hyperglycemia, and tissue lipid accumulation, accompanied with higher energy expenditure due to high expression levels of thermogenic genes in brown adipose tissue in HFD-fed lamp-2-deficient mice. Serum level of FGF-21 and its mRNA expression level in the liver were significantly higher in HFD-fed lamp-2-deficient mice in an ER stress-, but not PPAR-α-, dependent manner. These results suggest that a lamp-2-dependent fusion and degradation process of autophagosomes, and FGF-21 are involved in the pathogenesis of diabetes implicating a role for autophagy in this process (50). FGF activates phospholipases (51–53) that leads to the release of polyunsaturated fatty acid (PUFAs) that, in turn, can be utilized for the formation of various eicosanoids, LXs, resolvins, protectins, and maresins. Thus, PUFAs and LXs resolvins, protectins, and maresins could mediate anti-obesity and antidiabetic actions of FGFs. Alloxan, streptozotocin, and HFD block the activity of Δ^6^ and Δ^5^ desaturases and, thus, lead to a decrease in the synthesis and plasma and tissue levels of GLA, DGLA, AA, EPA, and DHA and decreased formation of LXs, resolvins, protectins, and maresins (from AA, EPA, and DHA) that could lead to increase in inflammation [increase in IL-6 and tumor necrosis factor-α (TNF-α)] and failure of resolution of inflammation and tissue repair. This may result in increase in peripheral insulin resistance, inflammation of mesenteric tissue, gut, adipose tissue, and liver (including NAFLD = non-alcoholic fatty liver disease). It may also lead to inflammation of hypothalamic neurons.

OK-432, an inducer of TNF-α, inhibited insulitis and autoimmune diabetes in NOD mice and BB rats that usually develop type 1 DM. Recombinant human (rh) TNF-α also blocks development of diabetes in NOD mice and BB rats. Administration of (5 × 10^4^ Units) rhTNF-α given by i.p. route twice a week to BB rats from 4 to 27 weeks of age prevented development of diabetes [non-treated rats showed 36.4% (8/22), whereas rhTNF-α-treated rats had 0% (0/21)] and the treated animals did not lose body weight, had normal blood glucose levels, and showed much less insulitis (45). These studies (39–41, 45) imply chronic and low dose and systemic administration of TNF-α and IL-2 regulate autoimmune diabetes in BB rats and NOD mice, suggesting that these animals may have a defect in TNF-α and IL-2-mediated immunoregulation. In an extension of these studies, it was observed that serum that contained TNF 75 U but not IL-1, IL-2, and IFN-γ (induced by OK-432 injection) when administered reduced the intensity of insulitis and inhibited the cumulative incidence of diabetes in NOD mice compared to the control. This inhibitory effect of the serum was diminished, although not significantly, by anti-mouse TNF antibody. OK-432-injected mice showed decreased Thy-1.2+ or CD8+ spleen cells and increased surface-Ig+ (S-Ig+) cells, whereas the proliferative response of spleen cells to concanavalin A (*P* < 0.01) and lipopolysaccharide (LPS) (*P* < 0.05) increased, suggesting that protections against the development of type 1 DM by OK-432 treatment in NOD mice was due to serum factors, including endogenous TNF and IL-2 (and probably some other unidentified factors) (46) (see Figures 1–3).

**Figure 4:**
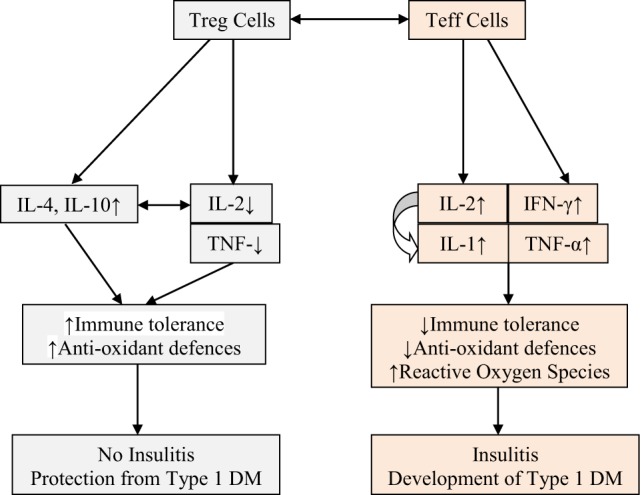
Scheme showing possible role of Treg and Teff cells and cytokines associated with these cells and their role in the prevention or development of type 1 diabetes mellitus (type 1 DM). Low-dose IL-2 and tumor necrosis factor-α (TNF-α) seem to prevent insulitis and development of type 1 DM by augmenting immune tolerance and enhancing antioxidant defenses in β cells. High levels of IL-2, TNF-α, and interferon-γ (IFN-γ) are pro-inflammatory in nature, enhance reactive oxygen species (possibly, NO) generation, decrease immune tolerance and antioxidant defenses of β cells, cause insulitis, and eventually lead to the development of type 1 DM (see text for further details). High levels of IL-2 enhance the production of interleukin-1.

Low-dose IL-2 selectively enhances IL-2-dependent STAT5 activation of Tregs in healthy individuals. In type 1 DM, IL-2 augments Tregs cells at an ~10-fold lower concentration of IL-2 than that is needed by T memory (T_M_) cells. This selective Treg activation responsiveness is due to their higher expression of IL-2 receptor subunit α (IL-2Rα) and γ chain and endogenous serine/threonine phosphatase protein phosphates 1 and/or 2 A activity. IL-2-dependent transcriptome in human Tregs is optimally activated by a 100-fold lower concentration of IL-2 in Tregs versus CD4^+^ T_M_ cells, implying that human Tregs possess an IL-2-dependent transcriptional amplification mechanism that selectively activates Tregs to induce their IL-2/IL-2R gene program. This explains as to why low-dose IL-2 therapy enhances Tregs for immune tolerance and its usefulness in type 1 DM (54, 55). These studies formed the basis of low-dose IL-2 therapy (0.33, 1, or 3 × 10^6^ IU/day. For *in vitro* studies, a dose of 1–10 IU/ml of IL-2 for 1 × 10^4^ each T cell subset is considered as low dose, whereas 100–1,000 IU/ml is considered as high dose). At low dose of IL-2, STAT5 will be activated only in Tregs that may have a role in preventing type 1 DM by restoring the unwanted immune responses to normal (56, 57) (see Figure 4).

In this context, it is noteworthy that toll-like receptor-4 (TLR-4) activation is believed to play an important role in islet cell inflammation and β cell loss in the development of type 1 DM. On the other hand, “hygiene hypothesis” suggests that bacterial endotoxin LPS, an agonist on TLR-4, inhibits type 1 DM. LPS administration to NOD mice during the prediabetic state delays the onset and decreases the incidence of type 1 DM. It is noteworthy that a multiple-injection protocol is more effective than a single LPS intervention in the prevention of development of type 1 DM. LPS-inhibited spleen T lymphocyte proliferation, augmented the generation of CD4(+)CD25(+)Foxp3(+) regulatory T cells (Tregs), decreased the synthesis of T-helper 1 (Th1) pro-inflammatory cytokines, and downregulated TLR-4 and its downstream MyD88-dependent signaling pathway. Multiple injections of LPS induced the development of tolerogenic dendritic cell (DC) subset that showed low TLR-4 expression with little influence on the DC phenotype. It is interesting that injection of dendritic cells (DCs) obtained from repeated LPS-treated NOD mice into NOD/SCID diabetic mice protected the progression of diabetes in the recipients. Thus, LPS prevents development of type 1 DM in NOD diabetic mice by Treg induction, downregulation of TLR-4 and MyD88-dependent signaling pathway possibly, by augmenting the development of a potential tolerogenic DC subset (47).

Based on the preceding discussion, it can be said that type 1 DM and other AID occur due to an imbalance between autoreactive effector T cells (Teffs) and regulatory T cells (Tregs). Till now, blocking Teffs with immunosuppression was considered as the only therapeutic approach, but now it is clear that activating/expanding Tregs seem to be a more attractive option without the toxicity of immunosuppression. It is also evident that low-dose IL-2 is safe to expand/activate Tregs in patients with type 1 DM. Low-dose IL-2 produced a dose-dependent increase in CD4(+)Foxp3(+) and CD8(+)Foxp3(+) Treg numbers and proportions. Tregs expressed higher levels of activation markers, such as CD25, GITR, CTLA-4, and basal pSTAT5, and showed a 20-fold higher sensitivity to IL-2 than Teff and NK cells. Furthermore, concentrations of regulatory cytokines in the plasma were increased in a dose-dependent manner, while cytokines linked to Teff and T helper 17 (Th17) inflammatory cells remained unchanged and Teff responses against β-cell antigens were suppressed. These results suggest that low-dose IL-2 therapy is useful in the prevention of type 1 DM and other autoimmune/inflammatory diseases (58) (see Figure 1).

## Alloxan, STZ-Induced Type 1 DM, IL-1, IL-2, and TNF-α, Free Radicals and PUFA Metabolism

There appears to be a strain-related susceptibility to the induction of type 1 DM by immune mediated toxins which could be correlated to the induction of high levels of IL-2, IFN-γ and TNF-α production. Macrophages are the first cells to infiltrate the islets in both multiple low-dose (MLD-STZ) induced type 1 DM in mice (59) and rats (60, 61) and BB rats that are prone to diabetes (62). It was reported that high levels of oxygen-free radicals produced by activated macrophages is seen in diabetes-prone BB rats prior to the appearance of inflammatory lesions in the islet cells (63). It was found that mRNA expression of Th1-type cytokines: IFN-γ, and IL-2 by infiltrating cells correlates with β-cell destruction and development of type 1 DM. Type 1 DM in NOD mice is promoted by Th1-type cytokines, while diabetes is prevented by T cells producing IL-4 and IL-10 (64). On the other hand, enhanced IFN-γ production increased susceptibility to MLD-STZ-induced type 1 DM; while downregulation of Th2 cells (and so decrease in the production of IL-4 and IL-10) downregulated the disease; and inhibition of IL-1 activity downregulated diabetes induction. Thus, mouse and rats’ susceptibility to develop STZ-induced type 1 DM and susceptibility of NOD and BB animals to develop diabetes is closely related to the higher levels of IL-2, IFN-γ, and TNF-α production by infiltrating macrophages and T cells and other cells. IFN-γ-mediated macrophage activation to produce pro-inflammatory cytokines, such as IL-2 and TNF-α, but not NO seems to be an important event in early diabetogenic action of invading immunocytes. Diabetes induction can be inhibited by suppressing IL-1 activity. Though NO seems to play a role in the development of type 1 DM, its extent of involvement and participation diabetes induction *in vivo* remains to be established (61, 65) (see Figure 1).

Pancreatic β cells are exquisitely sensitive to the toxic actions of reactive oxygen species (ROS). This is supported by the observation that autoimmune type 1 DM and alloxan and STZ-induced type 1 DM are associated with enhanced generation of free radicals (66–71). During the process of development of type 1 DM under various circumstances (autoimmune type 1 DM and chemical-induced type 1 DM) increased production of ROS and downregulation of antioxidant defenses, such as reduced glutathione (GSH) level and catalase, SOD, and thioredoxin (TRX), have been observed. This led to the suggestion that presence of adequate amounts of antioxidant defenses in β cells may protect them (β cells) and inhibit development of type 1 DM (72–75). This is supported by the observation that recombinant TRX protected β cells against apoptosis mediated through TNF and Fas pathways, and when overexpressed in β cells, TRX prevented development of type 1 DM in transgenic NOD mice (76). Furthermore, ALR/Lt, a NOD-related mouse strain that is resistant to alloxan-induced type 1 DM and autoimmune type 1 DM has elevated levels of systemic antioxidant defenses. In addition, ALR islets have almost fourfold elevated Kruppel-like factor 2 that upregulates antioxidant gene expression and inhibits NF-kB activation (77). It is known that NF-κB plays a key role in the cytokine-induced beta cell death (72). ALR islets that are resistant to alloxan-induced and autoimmune type 1 DM are not only cytokine resistant but have a defective nuclear translocation of NF-κB P65 subunit after cytokine treatment, which can be correlated to reduced kinetics of IκB degradation and suppressed iNOS induction (72). In contrast to this, β cells of NOD mice are exquisitely sensitive to cytokine-mediated apoptosis and are opposite to ALR islets that are resistant to cytokine and fee radical-mediated apoptosis (78). These results emphasize the significant role played by endogenous antioxidants in protecting pancreatic β cells against the cytotoxic actions of IL-1, TNF-α, IFN-γ, high levels of IL-2, and activated NF-kB that induce excess production of ROS and NO, Alloxan, and STZ also induce apoptosis of β cells by inducing enhanced production of ROS. In addition, there appears to be a deficiency of anti-inflammatory cytokines, such as IL-4 and IL-10, and an imbalance between Treg and Teff that paves way to apoptosis of β cells (see Figure 1). Of all, most important issue seems to be the cytotoxic action of high levels of IL-2 and β cell protective action of low-dose IL-2. These seemingly paradoxical actions of IL-2 suggest that in addition to its (IL-2) ability to restore the balance between Treg and Teff cells, low dose IL-2 can restore or enhance antioxidant defenses of β cells. In other words, high concentrations of IL-2 enhance free radical generation and reduce antioxidant content of β cells, whereas low concentrations of IL-2 has opposite actions. The big question is how different concentrations of IL-2 can produce these opposite actions. I propose that low and high concentrations of IL-2 have diametrically opposite actions on COX and LOX enzymes, IL-1 and metabolism of PUFAs (see Figures 2 and 3). These molecules, in turn, act on the gut microbiota, gut hormones, and hypothalamic neurotransmitters as detailed below.

## Interaction among IL-2, IL-1, COX, and LOX Metabolites and PUFAs

The fact that low-dose and high-dose IL-2 have differential action in the pathobiology of type 1 DM and Treg and Teff cells could be related to its interaction(s) with IL-1 and PGE2. For instance, (high dose) IL-2 increases the production of IL-1 and PGE2 (derived from AA) (79), which are pro-inflammatory molecules. On the other hand, PGE2 has a negative feedback control on IL-2 production (80–83). It is interesting to note that precursor of PGE2, AA and the precursor of AA, DGLA inhibit IL-1, IL-2, IL-6, and TNF-α production by themselves. AA and DGLA inhibit production of IL-1, IL-2, IL-6, and TNF-α production both in a PGE-dependent and PGE-independent manner (81–83). It is noteworthy that different doses of PGE2 show diametrically opposite actions on suppressor T (Treg) cells. A dose of 0.03–3.0 µM PGE2 did not show any suppressive action on cultures of spleen cells, whereas 3 nM PGE2 partially suppressed their proliferation. Surprisingly, indomethacin did not have any effect on this suppressor cell activity. On the other hand, alloantigen-activated proliferation of cells was inhibited by PGE2 in a dose-dependent manner. Influence of PGE2 on cell-mediated immunity seems to be directly proportional to its action on cell proliferation. Studies with indomethacin revealed that generation of suppressor cells is only partially dependent on PGE2 (84). The action of exogenous PGE2 on plaque-forming cell (PFC) response seems to depend on its time of action and dose. For instance, when PGE2 was added on Day 2 of the cultures, induction of the PFC response was inhibited, and the maximum inhibition (50%) was seen with 300 nM PGE2. On the other hand, when PBMs were cultured during the first 24 h with 300 nM PGE2 the PFC response was enhanced because of its action on T cells. Paradoxically, PGE2 when added on Day 0 did not affect the response though a prostaglandin-free monocyte supernatant rendered PGE2 suppressive. These results suggest that the monocyte supernatant had an inhibitory action on the stimulatory effect possibly, due to an interaction between PGE2 and T cells. These results imply that the actions of PGE2 depend on its time of action, the dose employed and its interaction with T cells and, possibly, other cells in the milieu (85).

It was reported that PGE2-sensitive T cells produce <200 pg/ml of both IL-2 and IL-4, while PGE2-resistant T cells secrete >1,000 pg/ml of IL-2, IL-4, or both. The involvement of IL-2 and IL-4 in these T cell responses was confirmed by the addition of exogenous lymphokines that restored PGE2-inhibited proliferation. By contrast, PGE2-resistant Th1-, Th2-, and Th0-like clones can be made PGE2 sensitive when IL-2, IL-4, or both were neutralized by the addition of antibodies to IL-2 and IL-4. These and other studies suggest that PGE2 predominantly suppressed CD45RA-RO + CD4 + T cells (Treg or suppressor cells) that secrete low levels of both IL-2 and IL-4 (86). In addition, PGE2 has also been shown to possess anti-inflammatory action (87, 88) by enhancing the production of IL-10. Furthermore, IL-2 when administered to patients with cancer produced a significant increase in IL-1 production *in vivo* that may account for some of its side effects (79). In this context, it is relevant to know about essential fatty acid metabolism and its relationship to the action of cytokines.

## Metabolism of Essential Fatty Acids (EFAs)

The dietary cis-linoleic acid (LA, 18:2 ω-6) and α-linolenic acid (ALA, 18:3 ω-3) are essential nutrients that need to be obtained in diet and, hence, are called as EFAs. LA is converted to gamma-linolenic acid (GLA, 18:3, ω-6) by the enzyme Δ^6^ desaturase. GLA is subsequently elongated to form di-homo-GLA (DGLA, 20:3, ω–6), the precursor of the 1 series of PGs. DGLA is acted upon by enzyme Δ^5^ desaturase to form arachidonic acid (AA, 20:4, ω-6), the precursor of 2 series of PGs, TXs, and the 4 series LTs. In a similar fashion, ALA is acted upon by Δ^6^ and Δ^5^ desaturases to form (EPA, 20:5, ω-3), the precursor of the 3 series of PGs and TXs and 5 series of LTs. EPA can be elongated to form docosahexaenoic acid (DHA, 22:6, ω-3). AA, EPA, and DHA can also form precursors to anti-inflammatory compounds: LXs, resolvins, protectins, and maresins. PUFAs and their metabolites, including PGs, LXs, resolvins, protectins, and maresins bind to G protein-coupled receptors on many cell types and mediate almost every stage of inflammation (89–92) (see Figure 3).

## LXs, Resolvins, Protectins, and Maresins

The two COX enzymes present in almost all cells and tissues are the constitutively expressed COX-1 and the inducible enzyme COX-2. Platelets are rich in thromboxane synthetase, which leads to the synthesis of TXA_2_ by platelets, a potent platelet-aggregator and vasoconstrictor. By contrast, vascular endothelial cells are rich in PGI_2_ synthetase and, hence, are capable of producing prostacyclin (PGI_2_), a vasodilator and platelet anti-aggregator. Endothelial cells have very low activity of thromboxane synthetase and so they produce very low amounts of TXA_2_. PGD_2_, PGE_2_, and PGF_2α_, which are major metabolites of the COX pathway, which possess pro-inflammatory actions.

There are 3 types of LOXs: 5-, 12-, and 15-LOXs (5-LO, 12-LO, and 15-LO). 5-LO is present in neutrophils, produces 5-HETE, a chemoattractant for neutrophils, that can be converted to LTs. LTB_4_ is a potent chemoattractant and activates neutrophils; whereas LTB_4_ induces aggregation and adhesion of leukocytes to vascular endothelium, and induces generation of ROS, and release of lysosomal enzymes. The cysteinyl-containing LTs C_4_, D_4_, and E_4_ (LTC_4_, LTD_4_, and LTE_4_) induce vasoconstriction, bronchospasm, and vascular permeability. LTs bring about their actions by binding to cysteiny leukotreine 1 and CysLT2 receptors.

Lipoxins (LXs) from AA; resolvins from EPA and DHA; protectins and maresins from DHA are generated that involves a transcellular biosynthetic mechanism involving neutrophils, platelets, and endothelial cells due to a complex yet collaborative effort among COX-2, 5-LOX, 12-LOX, and 15-LOX enzymes (93–98). LXs suppress leukocyte recruitment, neutrophil chemotaxis, and their adhesion to endothelial cells and possess a negative regulatory action on LT synthesis and action. By these actions, LXs can resolve inflammation. In general, an inverse relationship exists between LXs and LTs. Thus, the balance between LXs (and resolvins, protectins, and maresins) and LTs determines the degree of inflammation and its final resolution. In addition, LXs, resolvins, protectins, and maresins have potent anti-inflammatory and wound healing actions and, thus, function as endogenous anti-inflammatory and cytoprotective molecules. It is likely that defects in the synthesis and/or action of LXs, resolvins, protectins, and maresins may perpetuate inflammation in several inflammatory conditions (89–101).

## Anti-Inflammatory Cytokines IL-4 and IL-10 Enhance LXA4 Synthesis

IL-4 and IL-10 have been shown to enhance the conversion of AA, EPA, and DHA to their respective LXs (from AA), resolvins (from EPA and DHA), protectins (from DHA), and maresins (from DHA) indicating that this interaction between cytokines and bioactive lipids could be one of the principal mechanisms of their (IL-4 and IL-10) ability to suppress inflammation (99–104). IL-4 has been shown to upregulate 15-LO gene expression in human leukocytes that results in increased production of LXs. Glomeruli of experimental animals that showed spontaneous recovery from glomerulonephritis when injected with nephrotoxic serum showed higher levels of 12/15-LO mRNA and increased glomerular IL-4 mRNA, suggesting that T cell-derived IL-4 may regulate the expression of 12/15-LO during glomerulonephritis. These results suggest that IL-4 and LO interact with each other to initiate the recovery process from immune complex-mediated injury. Based on these pieces of evidence, it is suggested that LXA4, resolvins, protectins, and maresins are mediators of anti-inflammatory actions of IL-4 and IL-10 (99–104).

## The Balance Between LXA4 and LTS Determines the Degree of Inflammation

The 5-LOX action on AA (and on EPA and DHA) leads to the formation of LTs from infiltrating leukocytes, which are mediators of inflammation especially of experimental glomerulonephritis. LTB_4_ mediates and enhances neutrophil infiltration of target tissues (especially in glomerulonephritis), whereas LTC_4_ and LTD_4_ have vasoconstrictor actions that leads to a decrease in glomerular microcirculation. Blockade of the 5-LOX pathway ameliorated further deterioration of renal hemodynamic and structural parameters. By contrast, 15-S-hydroxyeicosatetraenoic acid (15-S-HETE), the immediate product of arachidonate 15-LOX, and LXs, produced by sequential 15- and 5- or 5- and 12-lipoxygenation of AA, generated during glomerular injury can antagonize leukotriene-induced neutrophil actions. LXA4 is a potent antagonist of LTD_4_ and LTC_4_ actions, especially, on the glomerular microcirculation. These contrasting effects of 5- and 15-LOX products ultimately influence the extent and severity of inflammation (105–108).

These results imply that IL-4 and IL-10 cytokines enhance the production of LXs, resolvins, protectins, and maresins and, thus, bring about their anti-inflammatory actions. This action of IL-4 and IL-10 may be in addition to their ability to suppress the production of pro-inflammatory IL-2, IL-6, TNF-α, MIF, and HMGB1 cytokines and LTs.

## Phospholipases, Pro- and Anti-Inflammatory Eicosanoids, Cytokines, and Their Relevance to Inflammation

**Figure 5:**
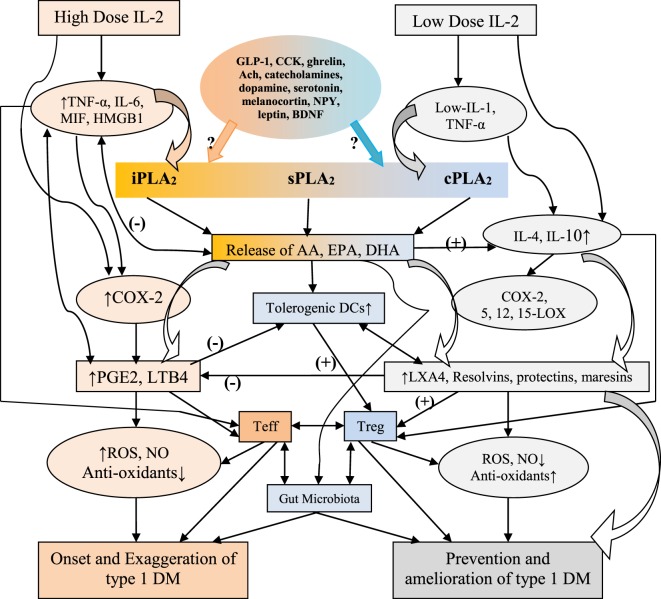
Scheme showing possible interaction among high and low doses of IL-2 in inducing and preventing type 1 diabetes mellitus (type 1 DM). It is proposed that high doses of IL-2 induce the activation of iPLA_2_ and COX-2 that leads to the synthesis and release of PGE2 and LTB4 and other pro-inflammatory molecules that enhance the formation of free radicals leading to apoptosis of pancreatic β cells and onset of type 1 DM. On the other hand, low doses of IL-2/tumor necrosis factor-α (TNF-α) activates sPLA_2_ and cPLA_2_ (cPLA_2_ > sPLA_2_) that leads to the formation of lipoxins, resolvins, and protectins; anti-inflammatory molecules, which decrease the formation of free radicals and enhance antioxidant capacity of pancreatic β cells and prevents type 1 DM.

There are three types of phospholipases that regulate AA and other PUFAs release: (i) calcium-independent PLA_2_ (iPLA_2_), (ii) secretory PLA_2_ (sPLA_2_), and (iii) cytosolic PLA_2_ (cPLA_2_). These three phospholipases have several isoenzymes. In the initial stages of inflammation, various PGs, LTs, and TXs are formed by the action of respective COX and LOX enzymes that induce exudate formation and inflammatory cell influx. Both PGE2 and LTB4 formations are triggered by the action of TNF-α, which can also initiate influx of neutrophils. By contrast, during resolution of inflammation LXA4, PGD2 and 15deoxyΔ^12-14^PGJ2 formation is increased. During the resolution phase of inflammation, a decrease in PGE2 synthesis occurs that is associated with decrease or complete absence of neutrophil influx and increase in phagocytosis of debris. Since PGE2 and LXB4 and LXA4 and PGD2 are derived from AA, it is evident that AA and other PUFAs are released in two phases: one at the onset of inflammation and the other during the resolution phase of inflammation. Thus, COX-2 enzyme participates both in pro-inflammatory (by increasing the formation of PGE2 and LTB4) and anti-inflammatory stages of inflammatory process (by increasing the formation of LXA4, resolvins, protectin, and maresins) [(98, 100, 101, 109–112) and see Figures 3 and 5].

It has been shown that from the initiation of inflammation up to 24 h, type VI iPLA_2_ protein expression is increased. On the other hand, from the beginning of 48–72 h type IIa and V sPLA_2_ expression is increased, whereas the expression of type IV cPLA_2_ that is not detectable during the early phase of acute inflammation is increased progressively during resolution phase of inflammation and peaking at 72 h. It is noteworthy that increase in type IV cPLA_2_ expression occurs in parallel with enhanced expression of COX-2 (112), implying that these enzymes are coupled to each other to regulate inflammation. These pieces of evidence suggest that different types of PLA_2_ have distinct and different roles in the inflammatory process. For instance, a reduction in the production of PGE_2_, LTB_4_, IL-1β, and platelet-activating factor (PAF) occurs when cPLA_2_ is inhibited. By contrast, inhibition of types IIa and V sPLA_2_ blocked PAF and LXA4 formation with a simultaneous reduction in the activities of cPLA_2_ and COX-2. These pieces of evidence suggest that sPLA_2_-derived PAF and LXA4 can enhance COX-2 and type IV cPLA_2_ expression and IL-1β induces the expression of cPLA_2_, suggesting that IL-1 has dual action: not only initiates and participates in the progression of inflammation but also plays a significant role in its resolution by enhancing the expression of cPLA_2_ (100, 101, 112–114). LXA4 suppresses the production of ILs that are induced by TNF-α; enhances TNF-α-mRNA decay, inhibits TNF-α secretion, and leukocyte trafficking and, thus, inhibits inflammation (96, 98, 100, 101, 115–120) (see Figures 3 and 5), suggesting that a close interaction exists between cytokines and bioactive lipids in the pathobiology of inflammation and its resolution process.

## PUFAs, LXA4, Resolvins, Protectins, and Type 1 DM

How can this knowledge about the pro- and anti-inflammatory actions of metabolites of various PUFAs be integrated to the pathogenesis of type 1 DM?

In a large population-based, case–control study, it was reported that supplementation of cod liver oil (a rich source of EPA and DHA) to pregnant women and/or children in their first year of life significantly lowered the risk of type 1 DM (28, 121). In addition, it was reported that incidence of type 1 DM is lower in those who have been breast fed for more than 3 months (human breast milk is rich in various PUFAs especially AA) (122). Brugman et al. reported that exclusive breast feeding delayed and partially protected bio-breeding diabetes-prone rats from type 1 DM (123) by enhancing the number of natural regulatory T cells [CD4(+) CD25(+) FoxP3(+)] in mesenteric lymph nodes and spleen not only after weaning period but also throughout life lending support to the beneficial action of cod liver oil supplementation in the prevention of type 1 DM. By contrast, stimulation of mesenteric lymph node cells from rats fed solid food during the nursing period showed enhanced production of IFN-γ, IL-4, and IL-10 compared to exclusive breastfed rats. Furthermore, exclusive breastfeeding increased the number of naturally occurring regulatory T (Treg) cells throughout life and decreased cytokine secretion at weaning (123). These results assume significance in the light of the fact that breast milk is not only a rich source of PUFAs but also contain significant amounts of LXA4, d-series resolvins (RvD1, RvD2, RvD3, AT-RvD3, and RvD4), protectin D1, maresin 1, and E-series resolvins (RvE1, RvE2, and RvE3) (124–128). PUFAs and anti-inflammatory metabolites of various PUFAs have immunological protective action that may be responsible for increased number of Treg cells noted in breast fed children by Brugman et al. and for the low incidence of type 1 DM in breast fed infants.

This relationship between PUFAs and their anti-inflammatory metabolites and type 1 DM is supported by the observation that in a mfat-1 transgenic mouse model whose islets contained increased levels of n-3 PUFAs and significantly lower amounts of n-6 PUFAs compared to the wild type, were resistant to apoptosis induced by TNF-α, IL-1β, and γ-IFN. The transgenic islets produced decreased amounts of PGE2, had reduced NF-kB activation and extracellular signal-related kinase 1/2 (ERK1/2) and enhanced pancreatic duodenal hemeobox-1 expression (129), events that render them resistant to the cytotoxic actions of. TNF-α, IL-1β, and γ-IFN. Fat-1 mice (fat-1 transgenic mice contain excess of n-3 PUFAs by converting n-6 AA to n-3 PUFAs) failed to show STZ-induced hyperglycemia (130) due to decreased production of TNF-α, IL-1β, and low NF-kB, and enhanced IkB pancreatic protein expression. In STZ-treated fat-1-animals, PGE2, and 12-hydroxyeicosatetraenoic acid (12-HETE) that are formed from AA were low and the anti-inflammatory LXA4 and 18-hydroxyeicosapentaenoic acid (18-HEPE), the precursor of the anti-inflammatory resolvin E1, were increased (130). These results are interesting since, despite the presence of increased tissue concentrations of n-3 PUFAs and low amounts of AA, in STZ-treated animals the pancreatic tissue showed elevated amounts of LXA4 and were resistant to diabetes. This is in support of our previous proposal that deficiency or low levels of AA and/or LXA4 occurs in subjects with DM and when their tissue levels are normal pancreatic β cells are resistant to the cytotoxic action of alloxan and STZ [(16–18) and see below]. In this study (130), the investigators reported enhanced production of resolvin E1 that they attributed to protection against development of STZ-induced DM.

In this study (130), though tissue and plasma content of AA is decreased, fat-1 mice showed enhanced formation of LXA4 treated with STZ. Both wild-type and fat-1 mice showed that the ratio between LXA4 and 18-HEPE (LXA4:18-HEPE) is ~5 (0:5—LXA4: 18-HEPE in wild type) and (8:40 pg/mg protein-LXA4:18-HEPE in fat-1 mice), respectively. Thus, near absence of LXA4 in wild type but its increase in fat-1 mice is interesting that may account for resistance of fat-1 mice to STZ-induced type 1 DM. It is likely that decreased or absence of LXA4 is responsible for the wild type to develop STZ-induced type 1 DM but not an increase in 18-HEPE. Thus, in fat-1 mice enhanced LXA4 formation despite decreased AA indicates that LXA4 prevents DM, whereas 18-HEPE is unlikely to be antidiabetic. In contrast to the results of the studies obtained with mfat-1 and fat-1 mice transgenic mouse models, we observed that AA is the most potent compared to other PUFAs in preventing alloxan-induced type 1 DM in Wistar rats (16–18). Since AA forms precursor to LXA4, these results are in support of the above argument that LXA4 is responsible for the decreased incidence of STZ-induced type 1 DM and in mfat-1 and fat-1 animal models.

**Figure 6:**
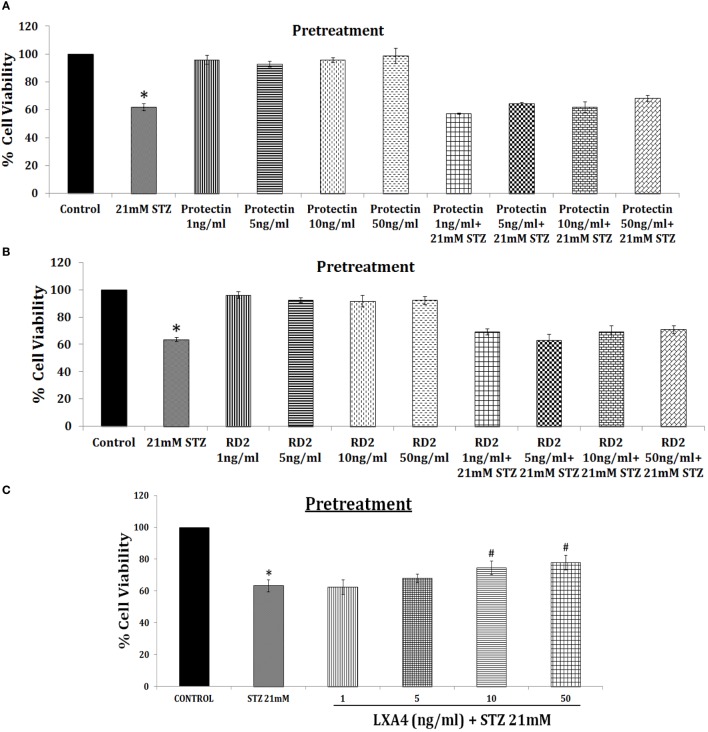
Effect of pre-treatment with resolvin D2 and protectin and lipoxin A4 (LXA4) on streptozotocin (STZ)-induced cytotoxicity to RIN5F cells *in vitro* [these data are taken from Ref. (137)]. **(A,B)** RIN5F cells were pretreated with 1, 5, 10, and 50 ng/ml of resolvin D2 and protectin, respectively to study its modulatory action on STZ (21 mM)-induced cytotoxic action. **(C)** RIN5F cells were pretreated with 1, 5, 10, and 50 ng/ml of LXA4 to study its modulatory action on STZ (21 mM) induced cytotoxic action. All values are expressed as mean ± SEM. *P ≤ 0.05 compared to untreated control, ^#^*P* ≤ 0.05 compared to STZ.

**Figure 7:**
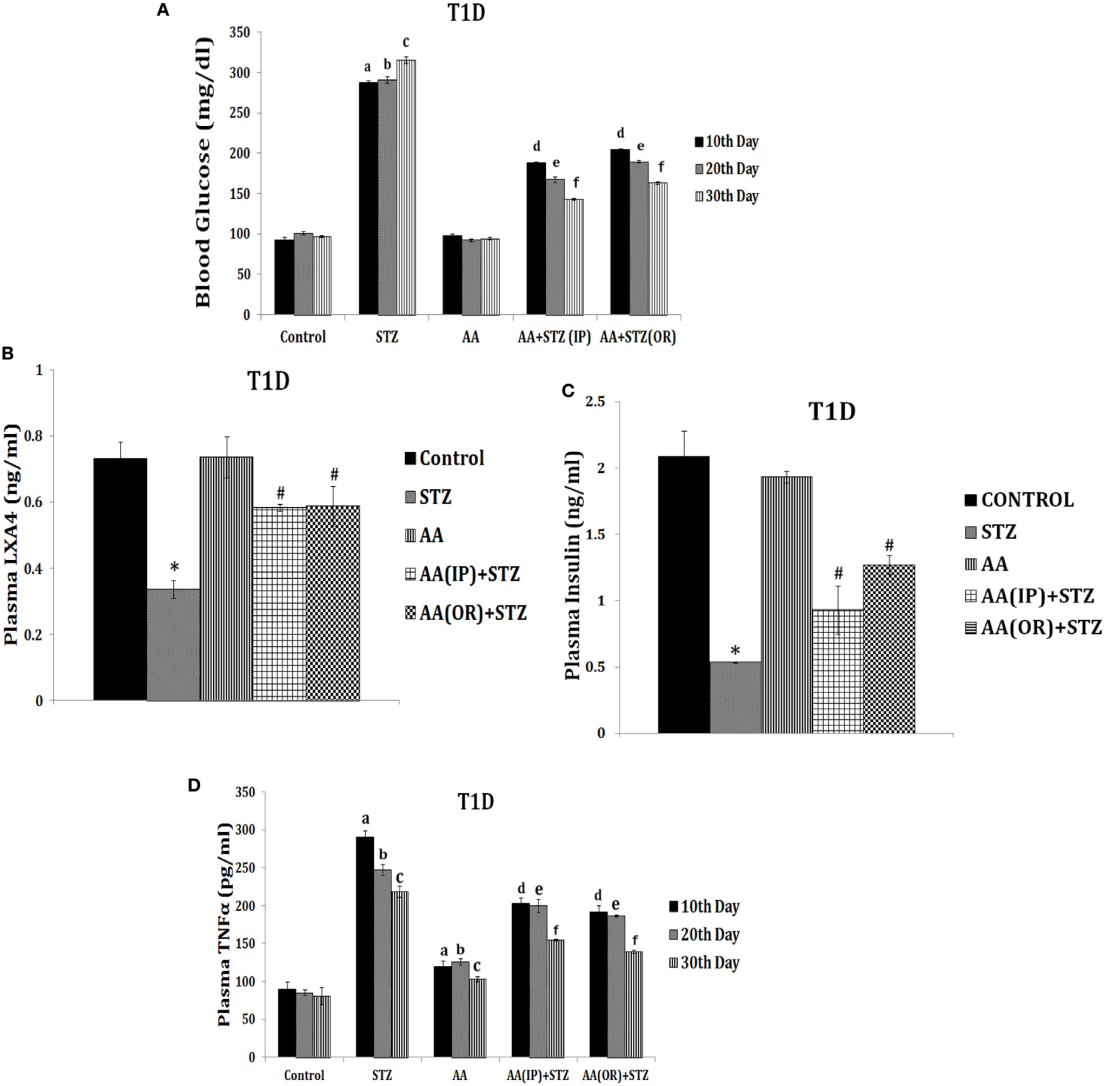
Effect of arachidonic acid (AA) on streptozotocin (STZ)-induced type 1 diabetes mellitus in Wistar rats [these data are taken from Ref. (137)]. These studies were approved by Institutional Animal Ethics committee. After 7 days of acclimatization, animals received 10 µg/ml of AA intraperitoneally (IP) or oral (OR) for 1 week and once in every week, whereas STZ 45 mg/kg of body weight was given only on day 1. **(A)** *Plasma blood glucose levels in animals*: blood glucose estimation was performed once in 10 days until the end of the study. All values are expressed as mean ± SEM. ^a^*P* ≤ 0.05 compared to 10th day control values. ^b^*P* ≤ 0.05 compared to 20th day control values. ^c^*P* ≤ 0.05 compared to 30th day control values. ^d^*P* ≤ 0.05 compared to plasma glucose levels seen on day 10 after STZ alone administration. ^e^*P* ≤ 0.05 compared to plasma glucose levels seen day 20 after STZ administration. ^f^*P* ≤ 0.05 compared to plasma glucose levels seen on day 30 after STZ administration. All the above set of experiments were done in triplicate on two separate occasions (*n* = 6) and values are expressed as mean ± SEM. *P ≤ 0.05 compared to untreated control. ^#^*P* ≤ 0.05 compared to STZ. **(B)** Measurement of lipoxin A4 levels in plasma of AA ± STZ treated animals at the end of the study (day 30). **(C)** Plasma insulin levels in AA ± STZ treated Wistar rats. Insulin estimation was done in the plasma collected at the end of the study. All values are expressed as mean ± SEM. *P ≤ 0.05 compared to untreated control. ^#^*P* ≤ 0.05 compared to STZ control (positive control group). **(D)** *Plasma tumor necrosis factor-*α* (TNF-*α*) level in AA *±* STZ treated rats*: TNF-α measurement was done in plasma collected once in every 10 days till the end of the study. All values are expressed as mean ± SEM. ^a^*P* ≤ 0.05 compared to the 10th day control; ^b^*P* ≤ 0.05 compared to the 20th day control; ^c^*P* ≤ 0.05 compared to the 30th day control; ^d^*P* ≤ 0.05 compared to the 10th day STZ control; ^e^*P* ≤ 0.05 compared to the 20th day STZ control; ^f^*P* ≤ 0.05 compared to the 30th day STZ control. *P ≤ 0.05 compared to untreated control; ^#^*P* ≤ 0.05 compared to STZ control. All values are expressed as mean ± SEM.

**Figure 8:**
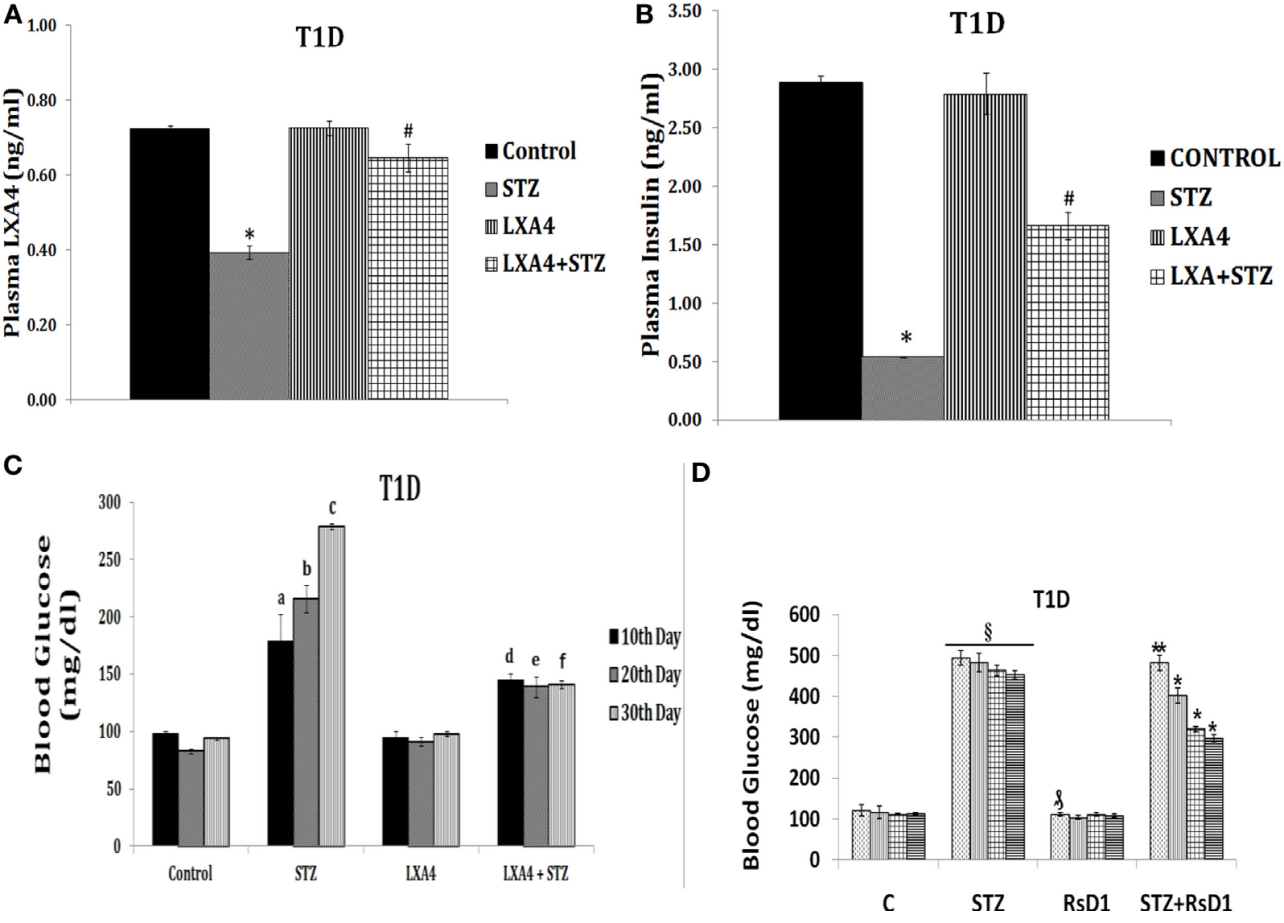
Effect of lipoxin A4 (LXA4) on streptozotocin (STZ)-induced type 1 diabetes mellitus (type 1 DM) **(A–C)** and resolvin D1 on STZ-induced type 1 DM **(D)** [these data are taken from Ref. (137) and unpublished data]. These studies were approved by Institutional Animal Ethics committee. T1D = type 1 DM. After 7 days of acclimatization, animals received 60 ng/ml LXA4 intraperitoneally for 5 days and 45 mg/kg body weight of STZ only on day 1. **(A)** *Plasma LXA4 levels measured on day 30 of the study*. **(B)** *Plasma glucose levels*: plasma glucose estimation was performed once in 10 days until the end of the study. All values are expressed as mean ± SEM. ^a^*P* ≤ 0.05 compared to 10th day control values; ^b^*P* ≤ 0.05 compared to 20th day control values; ^c^*P* ≤ 0.05 compared to 30th day control values; ^d^*P* ≤ 0.05 compared to 10th day STZ values; ^e^*P* ≤ 0.05 compared to 20th day STZ values; ^f^*P* ≤ 0.05 compared to 30th day STZ values; *P ≤ 0.05 compared to untreated control; ^#^*P* ≤ 0.05 compared to STZ control. All values are expressed as mean ± SEM. **(C)** *Plasma insulin levels*: plasma insulin levels were estimated on day 30. All values are expressed as mean ± SEM. *P ≤ 0.05 compared to untreated control; ^#^*P* ≤ 0.05 compared to STZ. **(D)** *Plasma glucose levels in STZ-induced type 1 DM treated with resolvin D1 (derived from DHA)*. *P < 0.05 compared to control.

This is further supported by our studies which revealed that LXA4 but not resolvins or protectins prevented alloxan and STZ-induced apoptosis of pancreatic β cells *in vitro* (unpublished data and see Figure 6), lending support to our contention that LXA4 is more efficient than resolvins and protectins in the prevention of type 1 DM. Furthermore, various PGs, thromboxane B2, and LTs were found to be less potent compared to AA in preventing alloxan-induced type 1 DM (131, 132). In an extension of this study, it was noted that both AA and its anti-inflammatory metabolite LXA4 prevented type 1 DM in Wistar rats, whereas both resolvin D2 and protectin were less effective (see Figures 7 and 8; unpublished data). It is evident from the data shown in Figure 7 that AA when given both orally and intraperitoneally completely prevented STZ-induced type 1 DM in Wistar rats. This protective action of AA against STZ-induced type 1 DM is accompanied by an increase in plasma LXA4 levels and decrease in plasma TNF-α levels, suggesting that AA possess anti-inflammatory actions by enhancing the formation of LXA4. In an extension of this study, we also noted that LXA4 by itself can prevent STZ-induced type 1 DM when given IP to Wistar rats (see Figure 8). It is interesting to note that plasma LXA4 levels were found to be increased on day 30 of the study (Figure 8). It is known that LXA4 has a very short half-life (only a few seconds to minutes). Despite this, even after 25 days after the last injection of LXA4 (LXA4 was given for 5 days and plasma levels were measured on day 30, whereas plasma TNF-α levels in the AA study was measured on days 10, 20, and 30) revealed that plasma LXA4 were increased in animals that were in receipt of the same. This suggests that the administered LXA4 is somehow stabilized and remained active till the end of the study (day 30), exogenous LXA4 stimulated endogenous production of LXA4 in an autocrine fashion or prevention of STZ-induced type 1 DM restored endogenous production of LXA4 to normal control values. In addition to its anti-inflammatory action, we noted that LXA4 increased the expression of PDX1 in RIN (rat insulinoma) cells *in vitro* (PDX1 is a homeobox protein expressed in β pancreatic cells that maintains and expresses the endocrine function of the pancreas) (unpublished data). Since, PUFAs and their metabolites may have a role in stem cell survival, proliferation, and differentiation (133–136), it is an intriguing possibility that AA and LXA4 (and possibly, resolvins, protectins, and maresins) may enhance proliferation of pancreatic β cells and/or augment proliferation and differentiation of pancreatic stem cells to insulin-secreting β cells. In this context, our recent study (see Figure 8D) revealed that intraperitoneal administration of resolvin D1 (60 ng/animal) to Wistar rats that were induced to develop type 1 DM by STZ did not show any change in plasma glucose levels by the end of first week. But at the end of second, third, and fourth weeks showed gradual decrease in plasma glucose levels to a significant degree. These results are surprising, indicating that resolvin D1 (and probably other similar compounds such as protectins and maresins), over a period, may gradually enhance the proliferation of residual β cells or induce proliferation and differentiation of pancreatic stem cells into β cells that can secrete insulin and ameliorate hyperglycemia. These interesting interpretations of our preliminary results (Figure 8D) need to be confirmed and established in future studies. These results are also interesting given the fact that LXs, resolvins, protectins, and maresins have very short half-life (from few seconds to minutes).

In addition, it is noteworthy that AA administered animals showed small but significant increases in plasma TNF-α levels (see Figure 7) compared to normal control but significantly less compared to the concentrations seen in STZ-administered animals. Thus, AA administered (oral or i.p.) seems to induce production of low concentrations of TNF-α (and possibly low IL-2) that, in turn, lead to Treg induction and enhanced the development of tolerogenic DCs and ultimately inhibition of type 1 DM.

Based on the preceding discussion and results obtained till date, the following is proposed. Low doses IL-2 and TNF-α activate sPLA2 and cPLA2 to induce the release of AA, EPA, and DHA that are converted to LXA4, resolvins, protectins, and maresins. PUFAs, LXA4, resolvins, protectins, and maresins enhance Treg formation and suppress that of Teff cells and stimulate synthesis of IL-4 and IL-10. It is expected that PUFAs, LXA4, resolvins, protectins, and maresins suppress production of ROS and enhance antioxidant defenses of pancreatic β cells that ultimately prevents development of type 1 DM. It is possible that PUFAs, LXA4, resolvins, protectins, and maresins stimulate formation and function of tolerogenic DCs (see Figures 1–5). These concepts are summarized in Figure 5. This is a simplified version of a complex set of interactions among cytokines, Treg, and Teff cells, PLAs, PUFAs and their metabolites, tolerogenic DCs, ROS, and antioxidants and pancreatic β cells. Nevertheless, this could form the basis for future studies to dissect the role of these factors in the pathobiology of type 1 DM.

By contrast, high doses of IL-2 stimulate production of excess of IL-1, TNF-α, IFN-γ, and HMGB1 (high mobility group box 1), activate iPLA2 that, in turn, can induce the release of AA, EPA, and DHA. These PUFAs lead to the formation of excess of PGE2, LTB4, and other pro-inflammatory bioactive lipids due to activation of COX-2, including excess generation of ROS, NO, and reduction in the antioxidant content in pancreatic β cells that will ultimately cause β cell apoptosis and development of type 1 DM (see Figure 5). Continuous activation of COX-2 and increased production of PGE2, LTB4, and ROS may result in decreased formation and activity of Treg and increase in the number of Teff cells and their activation; and deficiency of LXs, resolvins, protectins, and maresins which could result in apoptosis of β cells. Thus, an imbalance between cytoprotective and cytotoxic molecules/events results in the development of type 1 DM. The results of our studies that AA and LXA4 can prevent STZ-induced type 1 DM (see Figures 7 and 8) implies that cell membrane content of PUFAs and their response to various exogenous and endogenous stimuli, such as LPS, alloxan, STZ, IL-1, IL-2, TNF-α, diabetogenic viruses/bacteria, and so on, is an important event in the pathogenesis of type 1 DM. In this context, the role of gut microbiota in the pathobiology of type 1 DM needs attention.

## PUFAs and Gut Microbiota

It has been reported that gut microbiota has a significant role in the pathobiology of type 1 DM. It has been argued that the incidence of type 1 DM increased in recent years due to changes in the human microbial environment (138). For example, even though NOD mouse is used as a model of autoimmune DM or type 1 DM not all NOD mice (even though all are derived from a single diabetic female strain of mice) develop or express the same level of diabetes. This led to the suggestion that a transmissible environmental agent (possibly, gut microbiota) influences the incidence and severity of type 1 DM (139). Studies revealed that animal house microbial environment can influence the incidence of spontaneous type 1 DM in NOD mice (138). It is known that injection with Freund’s adjuvant or other various microbial products can decrease the incidence of type 1 DM (140, 141). This is supported by the observation that pathogen-free NOD mice lacking MyD88 protein (an adaptor for multiple innate immune receptors that recognize microbial stimuli) are resistant to the development of type 1 DM (34). It was reported that this effect is dependent on commensal microbes, implying that intestinal microbes interact with the innate immune system and modify development of type 1 DM (34). Subsequent studies revealed that development or protection from type 1 DM in NOD mice lacking MyD88 is dependent on the gut microbiota. These results suggest that both promotion and inhibition of autoimmunity can be performed by microbes by signaling through receptors such as TLRs (142). In general, it was noted that *Bacteroidetes* act in favor of protection from type 1 DM, whereas *Firmicutes* promote type 1 DM pathogenesis (143).

## How Gut Microbiota Prevent Type 1 DM?

Gut is the home to billions of both harmful and beneficial bacteria implying that the balance between these two forces determines gut health. It is clear from recent studies that gut microbiota determines not only gut health but also of other organs and systems as well. For example, it has been suggested that gut microbiota may have a role in the regulation of immune response, response to cancer therapy, neuronal function by regulating concentrations of various neurotransmitters, etc (34, 144–148). As already discussed above, development of type 1 DM can be influenced by gut microbiota and microbial products, such as LPS and Freund’s adjuvant (a product of mycobacteria). Wen et al. (34) reported that specific pathogen-free NOD mice lacking MyD88 protein are resistant to the development of spontaneous of type 1 DM. It was found that the composition of the distal gut microbiota changes due to MyD88 deficiency implying that the intestinal microbes interact with the innate immune system that modifies type 1 DM predisposition (34). How this exactly happens is not clear. It is likely that microbiota regulate gut and systemic immunocytes, produce metabolites that can act on the gut, gut-associated immunocytes, alter production and action of neurotransmitters, such as serotonin, both in the gut and hypothalamus in this cross talk between gut bacteria and pancreatic β cells.

For instance, microbes colonizing gut can induce and expand specialized Treg cells that prevent aberrant inflammatory responses to β cells and thus, maintain homeostasis. Recent studies (149, 150) revealed that a subpopulation of gut Treg cells express the nuclear hormone receptor retinoic acid receptor-related orphan receptor γt (RORγt) in response to microbiota-derived signals and, thus, control differentiation of TH17 cells and intestinal inflammation that may be relevant to induction of type 1 DM (151–154). Short-chain fatty acids, which are common bacterial metabolites, have been shown to selectively expand intestinal Treg cells (155) and increase RORγt-expressing Treg cells. Mice diet rich in the short-chain fatty acid butyrate also expand RORγt-expressing Treg cells (149). Furthermore, oral administration of the combination of 17 strains of *Clostridia* selected based on their high ability to enhance Treg cells abundance and inducing secretion of anti-inflammatory cytokine IL-10 and inducible T-cell co-stimulator in Treg cells from the human microbiota to adult mice-attenuated colitis and allergic diarrhea (156) and, possibly, this approach may also prevent type 1 DM. These results suggest that specific strains of useful bacteria may allow for tailored therapeutic manipulation of human immune disorders, including type 1 DM.

## Gut Microbiota and Serotonin

**Figure 9:**
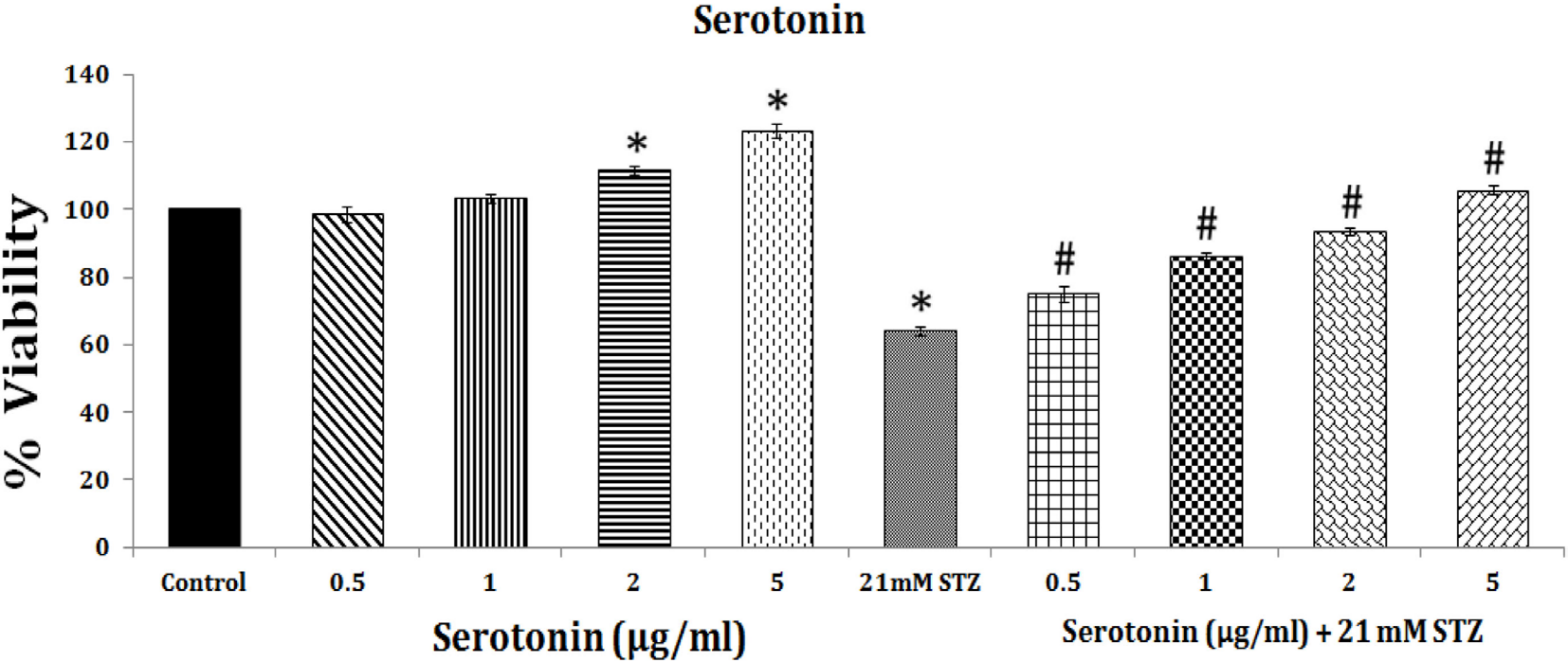
Effect of serotonin on the proliferation of RIN 5F cells *in vitro* and its modulatory effect on streptozotocin-induced inhibition (apoptosis) of RIN5F cells.

Gut microbiota depend for their nutrients on the food consumed by the individual to generate unique metabolites that, in turn, may provide the host unique nutrients that are likely to play a vital role in the regulation of immune development and immune response. This implies that gut microenvironment may influence the composition of the microbiota. Thus, by altering or alterations in the intake of dietary components, such as sugar, fat, or fiber, the energy sources for bacteria will be able to influence and determine which microbial species thrive in the gut. In a similar fashion, it is likely that alterations in host factors, including immunity influence the microbiota in the gut. In addition to their modulatory influence on immuno-inflammatory response as outlined above, gut microbiota also plays a critical role in regulating host serotonin production. Gut contains much of the body’s serotonin. Spore-forming bacteria from the mouse and human microbiota augment serotonin biosynthesis from colonic enterochromaffin cells (ECs), which supply serotonin to the mucosa, lumen, and circulating platelets (146, 147). It was noted that short-chain fatty acids acetate and butyrate elaborated by the gut microbiota determine enteric serotonin production, implying that gut microbiota influence the synthesis of serotonin by ECs and, thus, it (serotonin) may have an important role in beta cell function and proliferation. This is supported by the observation that during pregnancy there is an expansion of the maternal population of pancreatic β cells. Serotonin has been shown to act downstream of lactogen signaling to stimulate β cell proliferation. Inhibition of serotonin synthesis blocked β cell expansion. Thus, an integrated signaling pathway linking β cell mass to serotonin signaling pathway exists in the body (157, 158). These results indicate that serotonin pathway could be exploited to enhance β cells mass in those with type 1 DM. It is likely that exogenous and endogenous stimuli that reduce β cells mass in type 1 DM act by interfering with β cells mass enhancing ability of serotonin. In a recent study, we noted that serotonin can enhance the viability (obviously by enhancing proliferation) of rat insulinoma pancreatic β cells *in vitro* (see Figure 9). Thus, it is likely that serotonin not only enhances the viability and proliferation of pancreatic β cells by itself but is also capable of preventing apoptosis induced by STZ. This suggests that presence of adequate amounts of serotonin could increase the proliferation of β cells. Alternatively, delivery of serotonin to pancreas can, perhaps, increase the number of β cells and, thus, mitigate type 1 DM. Since gut microbiota metabolites, such as acetate and butyrate, enhance serotonin production from ECs, one mechanism by which gut microbiota prevent type 1 DM is by enhancing serotonin production that, in turn, increases the number of β cells.

**Figure 10:**
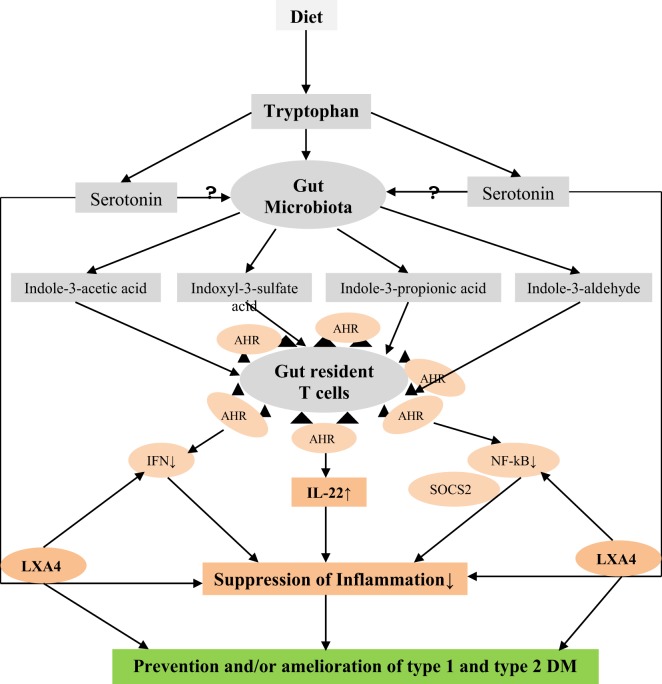
Scheme showing how tryptophan plays a role in the development of type 1 diabetes mellitus (type 1 DM) and type 2 DM. Tryptophan is an essential amino acid. Gut bacteria convert tryptophan into indole derivatives: indol-3-acetic acid, indoxyl-3-sulfate, indole-3-propionic acid, and indole-3-aldehyde that are ligands for the aryl hydrocarbon receptor (AHR). Tryptophan indole derivatives activate AHR in gut-resident T cells and innate lymphoid cells that produce IL-22, which protects against inflammation. Tryptophan metabolites by signaling through AHR influence a type 1 IFN signaling pathway that reduces NF-κB-driven inflammation (via SOCS2) and inhibits/ameliorates autoimmunity. Thus, tryptophan and its indole metabolites may have a role in autoimmune diseases (AID), such as type 1 DM and central nervous system AID. Tryptophan is also the precursor of serotonin that has immunomodulatory and cytoprotective actions (see Figure 9) and enhances lipoxin A4 production (unpublished data) and possibly that of resolvins, protectins, and maresins; anti-inflammatory bioactive lipids, which prevent type 1 DM (see Figure 8).

Tryptophan, an essential amino acid, present in the diet can be utilized by gut microbiota to form indole derivatives, such as indole-3-acetic acid, indoxyl-3-sulfate, indole-3-propionic acid, and indole-3-aldehyde, which are ligands for the aryl hydrocarbon receptor (AHR). It is known that activation of AHR of gut-resident T cells and innate lymphoid cells enhances production of IL-22, which protects against colitis. It is interesting that the susceptibility to colitis could be transferred to wild-type germ-free mice by transferring the microbiota (144). This two-way cross talk between microbes and the immune system may also be relevant to type 1 DM. The regulatory role of tryptophan in inflammatory response (159) is, in part, dependent on its conversion into AHR ligands by the microbiota (144, 159, 160). Tryptophan regulates the formation of neurotransmitter serotonin and this may link the role of serotonin in type 1 DM as discussed above. Thus, gut microbiota and their metabolites, tryptophan, serotonin, and β cell proliferation and inflammatory process, especially secretion of IL-22, are closely linked to each other in a complex fashion (see Figure 10). In addition, gut microbiota restrains excessive inflammation by promoting differentiation of Breg (B regulatory) cells in the spleen as well as in the mesenteric lymph nodes (161).

## PUFAs and Gut Microbiota

The purported role of PUFAs and their metabolites and gut microbiota in the pathobiology of type 2 DM and metabolic syndrome implies that there could occur a close interaction between them. This is supported by the observation that supplementation of (LA, 18:2 n-6) and CLA to two individuals leads to the formation of vastly different products though are metabolized at similar rates. This difference in the products formed has been attributed to the action of colonic bacteria. It was reported that proportion of propionate and butyrate formed were higher in those who contained mainly *Bacteroidetes* (54% of clones), implying that products formed from ingested lipids and other dietary constituents in the intestine of different individuals may depend on the gut microbiota profile that, in turn, may have significant impact on gut health. In addition, higher concentrations of the n-3 EPA and DHA were noted in the adipose tissue of those who were fed their precursors and was also associated with reductions in the pro-inflammatory TNF-α and IFN-γ, suggesting that the metabolome is a composite of host and microbiota metabolic activity (162).

These results are particularly interesting since under various pathological situations especially in many genetic or infectious diseases (including AID, such as lupus and inflammatory bowel disease) the balance between host and microbiota may be altered that leads to erroneous communication resulting in significant changes in the composition of the human and gut metabolome. This may explain changes in the level of hydroxy, branched, cyclopropyl and unsaturated fatty acids, aldehydes, and phenyl derivatives in blood of patients with various diseases (163, 164). One such notable change could be in the formation of branched fatty acid esters of hydroxy fatty acids, such as palmitic-acid-9-hydroxy-stearicacid whose formation is regulated by fasting and high-fat feeding. PAHSA increases insulin sensitivity and its administration lowered plasma glucose levels by stimulating glucagon-like peptide-1 (GLP-1) and insulin secretion and reducing adipose tissue inflammation (164). These studies suggest that human gut microbiota metabolome include compounds such as PAHSA (and other hydroxy fatty acids) that have the potential to suppress insulin resistance and ameliorate type 2 DM.

## Gut Microbiota, Endocannabinoid System, and Obesity

In addition, obesity is characterized by increased endocannabinoid system tone and endocannabinoid system controls gut permeability and adipogenesis. Gut microbiota selectively modulates colonic CB1 mRNA expression (165). Anandamide (AEA) and 2-arachidonoylglycerol (2-AG), the endogenous CB1 and CB2 ligands, and fatty acid amide hydrolase and monacylglycerol lipase, the main enzymes responsible for their degradation, respectively, can be affected by gut microbiota in the colon but not in the jejunum. Intestinal AEA and 2-AG tissue content and plasma LPS are reduced in genetically obese mice fed prebiotics. CB1 receptor antagonist treatment decreased adiposity, blood glucose levels, and gut permeability and inhibited the expression of hepatic inflammatory markers TNF-α, PAI-1, and TLR4 mRNA. Thus, gut microbiota participates in the development of adipose tissue. Prebiotics decrease AEA levels lending support to the concept that gut microbiota modulate the endocannabinoid system (166).

Dietary EPA and DHA can modulate endocannabinoid synthesis. EPA and DHA can displace AA from phospholipid membranes and reduce AEA and AEA synthesis and enhance the formation of eicosapentaenoyl ethanolamide and docosahexaenoyl ethanolamide (167–171), which, in turn, decrease pro-inflammatory adipocyte IL-6 and monocyte chemotactic protein-1 production (172).

It is evident from the preceding discussion that the CB1 receptor and its endogenous ligands, AEA and 2-AG, control energy balance by influencing lipid and glucose metabolism (173).

**Figure 11:**
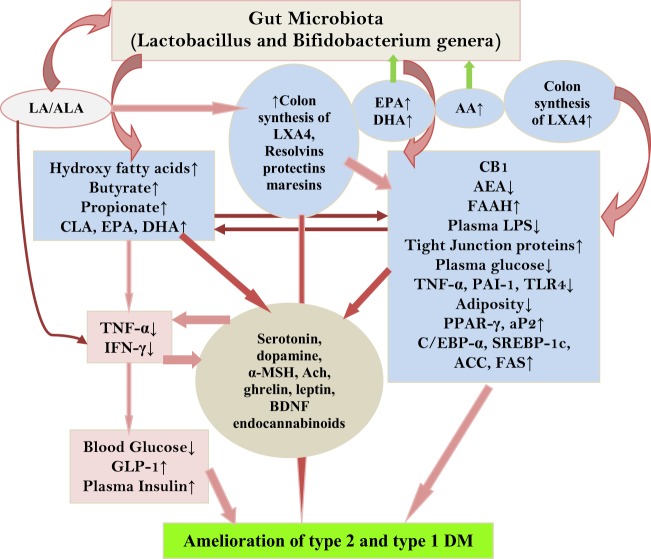
Scheme showing possible interaction(s) among gut microbiota, gut microbial metabolites, polyunsaturated fatty acid (PUFAs) and their metabolites [lipoxin A4 (LXA4), resolvins and protectins], and endocannabinoid system. It is possible that endocannabinoid receptors in the hypothalamus and other brain areas also play a role in DM. A stronger role for endocannabinoid system is seen in type 2 DM compared to its role in type 1 diabetes mellitus. It is likely (needs firm evidence) that gut microbiota metabolizes dietary linoleic acid and α-linolenic acid to arachidonic acid (AA) and eicosapentaneoic acid (EPA) and docosahexaenoic acid (DHA), respectively, that may enhance the formation of anti-inflammatory and antidiabetic molecules LXA4 (from AA), resolvins (from EPA and DHA), and protectins (from DHA, see Figures 7 and 8 also). AA, EPA, and DHA may enhance the proliferation of useful microbiota. Thus, there could be a two-way interaction between PUFAs and gut microbiota. PUFAs have antibiotic-like actions and so may suppress the proliferation of harmful bacteria that are associated with obesity. There could be an interaction between endocannabinoid system, gut microbiota metabolites, and hypothalamic neurotransmitters as shown in the figure.

Though it is not yet clear, it is possible for an interaction(s) among gut microbiota, gut microbial metabolites, PUFAs, and their metabolites (LXA4, resolvins, protectins, and maresins), and endocannabinoid system as shown in Figure 11. It is likely that endocannabinoid receptors in the hypothalamus and other brain areas also play a role in DM. A stronger role for endocannabinoid system is seen in type 2 DM compared to its role in type 1 DM. It is likely that gut microbiota converts dietary LA and ALA to their respective AA and EPA and DHA, respectively, that, in turn, may lead to an increase in the formation of their anti-inflammatory and antidiabetic molecules: LXA4 (from AA), resolvins (from EPA and DHA), and protectins and maresins (from DHA, see Figures 7 and 8 also), especially in the colon. There is evidence to suggest that endocannabinoid system does influence serotonin and dopamine release and action (167–170, 174–176) implying an interaction between these two systems (see Figure 11) that may be relevant to their involvement in the pathobiology of type 1 DM.

## Type 2 DM

Type 2 DM that accounts for more than 90% of those with diabetes is characterized by peripheral insulin resistance and low-grade systemic inflammation. Patients with type 2 diabetes are generally obese, show insulin resistance, especially those of South East Asians descent (especially in persons of Indian subcontinent), have an increased percentage of body fat in the form of abdominal obesity, and have intra- and inter-myocellular and β cell triglyceride accumulation. Type 2 diabetes often shows strong genetic predisposition but is not well defined.

## Pathobiology of Type 2 Diabetes

The exact cause for type 2 diabetes is not clear. Since, there is no insulin deficiency, at least in the early stage of diabetes, it is obvious that pancreatic β cells are not at fault; but this implies that islet cells are not able to secrete enough insulin to overcome peripheral insulin resistance in these patients. In other words, if peripheral insulin resistance is corrected, then probably insulin secreted by β cells is adequate to maintain normoglycemia. Low-grade systemic inflammation plays a significant role in the onset of type 2 DM (171, 177–182) since these patients have an increase in the plasma levels of C-reactive protein (CRP), TNF-α, IL-6, and resistin, whereas the concentrations of adiponectin are reduced (182–191).

## Low-Grade Systemic Inflammation and Type 2 DM

Elevated plasma CRP, TNF-α and IL-6 levels can induce endothelial dysfunction (183, 186–192) by enhancing free radical generation and simultaneously reducing endothelial nitric oxide (eNO) generation and its half-life (193–198). We have demonstrated that plasma eNO levels are low in those with type 2 DM (183, 189, 190).

It is not known whether inflammation precedes or is secondary to the development of type 2 diabetes (197, 198). IL-6 and TNF-α increase neutrophil superoxide anion generation (193, 194) that can inactivate eNO and prostacyclin (PGI_2_) resulting in endothelial dysfunction. By contrast, adequate production of eNO inactivates ${\text{O}}_{2}^{-}$ and, thus, prevents/arrests thrombosis and atherosclerosis (194–199). Thus, enhanced oxidative stress could be one factor that contributes to the development of type 2 DM.

Adipose tissue is the source of several soluble factors, such as adiponectin, resistin, and corticosterone. Adiponectin has anti-inflammatory actions and its plasma levels are inversely correlated to insulin resistance, whereas resistin enhances insulin resistance (200–203), and has pro-inflammatory actions (204, 205). Transgenic mice over expressing adipose tissue 11 β-hydroxysteroid dehydrogenase type 1 (11β-HSD-1) developed abdominal obesity and showed several features of type 2 DM, such as insulin resistance, hyperlipidemia, and hyperphagia (206), which led to the suggestion that type 2 DM could be termed as “localized Cushing’s syndrome.”

Based on these pieces of evidence, it is possible to use elevated plasma concentrations of CRP, IL-6, resistin, TNF-α, and reduced levels of NO and adiponectin as markers to predict the development and progression of type 2 DM, hypertension, and coronary heart disease (207–210), whereas reductions in their levels (CRP, IL-6, and TNF-α) and increase in those of adiponectin and NO induced by diet control, exercise, and statin therapy may suggest a better prognosis in these patients. Hence, it may be worthwhile to measure these pro- and anti-inflammatory markers to predict the development of type 2 DM and its response to various therapies.

## Nitric Oxide and Type 2 DM

Nitric oxide is synthesized from l-arginine by three different types of nitric oxide synthases (NOS)—the endothelial (eNOS), the neuronal (nNOS), and the iNOS (211, 212). It is noteworthy that eNOS and nNOS are constitutively expressed, whereas iNOS is induced in macrophages and other cells by TNF-α and IFN-γ.

Nitric oxide, a vasodilator, platelet anti-aggregator, and an inhibitor of vascular smooth muscle cell proliferation is also capable of regulating leukocyte recruitment. eNO prevents leukocyte rolling and adhesion to postcapillary venules. Thus, physiological concentrations of NO inhibit inflammation, while its high levels as seen in inflammatory conditions produces harmful actions (177, 213–223). NO may be converted to peroxynitrite radical that is pro-inflammatory in nature. Insulin resistance, obesity, atherosclerosis, diabetes, and hypertension are associated with decreased eNO production (177, 215–219). NO and its derivatives are microbicidal and, thus, NO serves as an endogenous host defense mediator against infections (213).

## ROS and the Interactions Between ROS and NO and Their Role in Type 2 DM

Reactive oxygen species or oxygen-derived free radicals produced by leukocytes, macrophages, and other similar cells in response to exposure to various stimuli and following a phagocytic challenge (220–223) occurs because of activation of the NADPH oxidative system. ROS species: superoxide anion $\left({\text{O}}_{2}^{-}\right)$, hydrogen peroxide (H_2_O_2_), and hydroxyl radical (OH) react with NO to form reactive nitrogen intermediates (RNI) that are cytotoxic to various organelles of cells (213, 220–223). ROS and RNI even at very low concentrations can increase the expression of chemokines (e.g., IL-8), cytokines, and endothelial leukocyte adhesion molecules that, in turn, amplify the inflammatory cascade (220–223). ROS and RNI induce endothelial cell damage and increase vascular permeability, cause insulin resistance, and produce thrombus formation. Activated adherent neutrophils not only produce ROS and RNI but are also capable of stimulating xanthine oxidase in endothelial cells. This further aggravates superoxide anion generation. ROS and RNI inactivate α_1_-antitrypsin resulting in unopposed protease activity that results in increased destruction of extracellular matrix. Type 2 DM is characterized by endothelial dysfunction and increased generation of ROS is seen in this condition. It is likely that hyperglycemia and insulin deficiency may stimulate ROS generation and decrease eNO generation in type 2 DM (224–226) and this explains as to why strict control of plasma glucose levels is beneficial.

Production of physiological concentrations of eNO occurs only when endothelial cells are healthy. In view of this, plasma concentrations or endothelial production of NO may serve as a marker of endothelial cell integrity and health. Plasma concentrations of eNO are low in subjects with type 2 DM, suggesting that endothelial dysfunction is present in them. These reduced levels of eNO levels revert to normal following weight loss, implying that plasma eNO levels could be used as a marker of endothelial function and to judge adequacy of treatment.

## Insulin Resistance and Type 2 DM are Common in Indians (South East Asians): Why and How?

Indians (South East Asians) compared to Western population have a higher incidence of abdominal obesity, high prevalence of type 2 DM, hypertension, low concentrations of high-density lipoprotein (HDL) cholesterol, hypertriglyceridemia, and hypercholesterolemia, all of which are associated with insulin resistance (227–239). Hyperinsulinemia may be a consequence of higher body fat or abdominal obesity in Indians even when body mass index (BMI) is normal that may explain the presence of insulin resistance and hyperinsulinemia (227–231) in them.

In this context, it is interesting to note that mice over expressing enzyme 11β-hydroxysteroid dehydrogenase type 1 (11β-HSD-1) selectively in adipose tissue develop abdominal obesity show insulin resistance, hyperlipidemia, and hyperphagia despite hyperleptinemia (206, 232–235), features that are seen in Indians with obesity and type 2 DM, though this has been disputed (235). Previously, I proposed that higher activity of 11β-HSD-1 will be higher in the abdominal adipose tissue of Indians compared to Caucasians (230) that may explain why abdominal obesity, insulin resistance, and type 2 DM are common in Indians.

In a similar fashion, Pima Indians, who have high incidence of insulin resistance and type 2 DM, were documented to show a positive correlation between adipose 11β-HSD1 activity and total (BMI, percentage body fat) and central (waist circumference) adiposity and fasting glucose, insulin, and insulin resistance. Intra-adipose cortisol was positively associated with fasting insulin but not with 11β-HSD1, suggesting that higher adipose 11β-HSD1 activity is associated with features of the metabolic syndrome (233). It was observed (234, 235) that 11β-HSD1 mRNA levels were higher in omental compared with subcutaneous preadipocytes.

Indians have higher plasma levels of pro-inflammatory markers such as CRP than do the Caucasians (236–238) that could be related to the presence of metabolic syndrome seen in them (236–240). HDL is known to stimulate eNO synthesis (241), whereas NO, in turn, can inhibit LDL oxidation (242, 243). Oxidized LDL and decreased levels of eNO indicate a high risk for atherosclerosis and thrombosis, which may explain higher incidence of coronary heart disease in Indians. This assumption is supported by the results of our study wherein we demonstrated the occurrence of high levels of plasma lipid peroxides and low NO concentrations in Indians with type 2 diabetes (244). TNF-α and IL-6 augment whereas insulin-like growth factor-I and insulin inhibit 11β-HSD-1 (245–249) activity, while insulin and IGFs suppress TNF-α and IL-6 and enhance eNO synthesis, implying a close interaction among cytokines, eNO, and insulin. This also explains the anti-inflammatory actions of insulin (244, 250).

Since insulin has anti-inflammatory actions (244, 250–258), it is tempting to speculate that one of the functions of hyperinsulinemia is to suppress low-grade systemic inflammation. In addition, leptin is pro-inflammatory in nature (259). Indian children (including Pima Indians) have hyperinsulinemia and hyperleptinemia compared to their Caucasian counterpart (260–262), suggesting that insulin resistance and low-grade systemic inflammation are present in them from early life.

It is likely that increased 11β-HSD-1 activity of adipose tissue in Indians is because of increased plasma and tissue levels of pro-inflammatory cytokines, such as CRP, IL-6, and TNF-α (230–235) that may be responsible for the high incidence of abdominal obesity in Indians. Insulin, IGFs, TNF-α, and IL-6 regulate 11β-HSD-1 activity in adipose tissue. Hence, the final expression of 11β-HSD-1 in abdominal adipose tissue depends on the balance between pro- and anti-inflammatory molecules (TNF-α and IL-6 versus insulin and IGFs) (245–249). Thus, presence of abdominal obesity is a physical sign of elevated levels of TNF-α, IL-6, lipids peroxides (since these cytokines stimulate free radical generation), LDL, oxidized LDL, hyperleptinemia, hypertriglyceridemia, and resistin; and low levels of HDL, eNO, adiponectin, IL-4, IL-10, and insulin resistance; hyperinsulinemia and glucose intolerance. This may explain the high incidence of type 2 DM in South East Asians and Pima Indians.

## Is Type 2 DM Having Its Origins in the Perinatal Period?

Type 2 DM may have its origins early in life since low birth weight newborn showed high prevalence of type 2 DM in later life (263–266). Indian babies are small, who were 2.95 kg or less at birth compared to Western infants whose birthweight was more than 4.31 kg. Type 2 DM and metabolic syndrome are 10 times greater in those who were 2.95 kg or less at birth.

## ω-3 and ω-6 PUFAs in Type 2 DM and Insulin Resistance

For adequate fetal growth ω-3 and ω-6 PUFAs are essential (267–274). Preterm infants have decreased activity of Δ^6^ and Δ^5^ desaturases and so form low amounts of EPA, DHA, and AA. AA status correlated with measures of normalized growth through 12 months in infants and it improves first year growth of preterm infants (272) by stimulating glucose uptake by cells (267, 272). On the other hand, EPA and DHA prolong gestation and/or increase fetal growth rate and, thus, contribute to increase in birth weight (273, 274). Some of the actions of PUFAs that are relevant to the present discussion are the following.

1. EPA and DHA inhibit TNF-α and IL-6 production that accounts for their anti-inflammatory actions.
2. EPA, DHA, and AA enhance eNO generation.
3. EPA, DHA, and AA inhibit 3-hydroxy-3-methylglutaryl coenzyme A (HMG-CoA) reductase activity and, thus, regulate cholesterol metabolism, suggesting that PUFAs function as endogenous statins (275–279).
4. PUFAs are endogenous ligands for PPAR-α and PPAR-γ and, thus, they have actions like thiazolidinediones. PPARs have anti-inflammatory actions by virtue of their ability to suppress TNF-α and IL-6 production and inhibit free radical generation; and at the same time, they enhance the production of adiponectin. By functioning as ligands of PPARs, PUFAs augment adiponectin production and prevent/arrest atherosclerosis.
5. EPA, DHA, and AA ameliorate insulin resistance. EPA and DHA ameliorated insulin resistance and hypertension in experimental animals. EPA reduced insulin resistance and decreased the incidence of type 2 DM in experimental animals. Insulin sensitivity correlated with concentrations of EPA, DHA, and AA in skeletal muscle phospholipids in humans (275, 280–290). Furthermore, a recent study showed that unsaturated fat improves insulin resistance and oxidative stress status in subjects with abdominal obesity in postprandial state (291).
6. EPA and DHA suppress leptin gene expression (292). Leptin has pro-inflammatory actions. EPA/DHA functions as endogenous anti-inflammatory molecule by inhibiting leptin production.
7. Indians (who as a race are at high risk of metabolic syndrome including hypertension) showed significantly lower plasma concentrations of AA, EPA, and DHA compared to healthy Canadians and Americans (293).
8. Higher levels of PUFAs in the cell membranes enhances the number of insulin receptors on the membrane and their affinity to insulin by rendering membrane more fluid and thus, decreases insulin resistance. By contrast, saturated fatty acids have opposite actions and increase insulin resistance (294).

These pieces of evidence led us to suggest that insulin resistance and type 2 DM are common in Indians because of perinatal deficiency of EPA, DHA, and AA. Maternal protein restriction and/or increased consumption of saturated and/or trans-fatty acids and energy rich diet during pregnancy may lead to a decrease in the activity of Δ^6^ and Δ^5^ desaturases, which are needed for the conversion of dietary EFAs: LA and ALA to their respective long-chain PUFAs (see Figure 4 for metabolism of EFAs). This ultimately results in both maternal and fetal deficiency of EPA, DHA, and AA. Perinatal protein depletion causes significant decrease in the activities of Δ^6^ and Δ^5^ desaturases in fetal liver and placenta (295). Thus, activities of Δ^6^ and Δ^5^ desaturases are decreased by both protein deficiency and high-energy diet intake.

Eicosapentaneoic acid, DHA, and AA are known to inhibit TNF-α and IL-6 synthesis. This implies that high plasma levels of TNF-α and IL-6 seen in instances of insulin resistance is due to EPA, DHA, and AA deficiency. Thus, maternal and fetal deficiency of EPA, DHA, and AA tends to increase the plasma and tissue levels of TNF-α and IL-6 in the fetus. This may explain why prenatal exposure to TNF-α produces obesity (296), and obese children and adults have high levels of IL-6 and TNF-α (240, 297). Low plasma and tissue concentrations of PUFAs such as EPA, DHA, and AA can decrease secretion of adiponectin (298, 299) that can aggravate insulin resistance and enhance the chances of development of type 2 DM. Increased circulating levels of TNF-α and IL-6 augment the activity of 11β-HSD-1 resulting in an increase in the production of corticosterone in the adipose tissue, which can lead to abdominal obesity characteristically seen in Indians and those with insulin resistance and type 2 DM.

## Ventromedial Hypothalamic (VMH) and Type 2 DM

Hypothalamus plays a significant role in the control and maintenance of plasma glucose and insulin secretion, indicating that stimuli or insults induced during the growth of brain during the perinatal period may play a major role in the pathogenesis of diabetes. Thus, hormonal and/or nutritional factors acting during the perinatal period and early childhood may have lifetime consequences and program the development of insulin resistance, obesity and type 2 DM in later life (300–303). In experimental animals, VMH lesion may induce hyperphagia and excessive weight gain, fasting hyperglycemia, hyperinsulinemia, hypertriglyceridemia, and impaired glucose tolerance, all features of metabolic syndrome. In these animals, intraventricular administration of antibodies to neuropeptide Y (NPY) abolished hyperphagia. STZ-induced diabetic animals have increase in NPY concentrations in their paraventricular, VMH and lateral hypothalamic area, whereas VMH-lesioned rats show decreased concentrations of norepinephrine and dopamine in the hypothalamus. By contrast, long-term infusion of norepinephrine and serotonin into the VMH impaired pancreatic islet cell function. These abnormalities in the hypothalamic neurotransmitters revert to normal after insulin therapy, indicating that dysfunction of VMH impairs pancreatic β-cell function and induces metabolic abnormalities that are consistent with type 2 DM (304–308). It is true that other hypothalamic nuclei also play a significant role in energy homeostasis, obesity, and type 2 DM (309–313). In this context, it is noteworthy that several hypothalamic peptides and monoamines modulate inflammation (313).

## Hypothalamic Peptides and Neurotransmitters as Immunomodulators and Regulators of Inflammation

### Dopamine

Several studies suggest that dopamine has anti-inflammatory actions. For instance, apart from being a neurotransmitter dopamine induced polymorphonuclear leukocyte (PMNL) apoptosis and modulated its function (314), reduced PMNL migration, suppressed PMNL CD11b/CD18 and E-selectin and ICAM-1 expression, and interaction between PMNLs and the endothelium. In addition, studies suggested that dopamine induced splenocyte apoptosis, decreased splenocyte proliferation and IL-2 and IFN-γ release in mice (313, 315). Obese subjects have decreased dopamine receptors and decreased dopamine levels in the brain (316) and are considered to have “reward deficiency syndrome.” A decrease in the dopamine receptor number or content in the brain of obese subjects is in support of the observation that low-grade inflammation may occur in the hypothalamus and cause its dysfunction that ultimately lead to the development of type 2 DM and metabolic syndrome (313).

### Serotonin

Like dopamine, serotonin and its precursor, 5-hydroxy-l-tryptophan, suppressed T-cell-dependent, humoral, hemolytic, primary immune response in mice, reduced thymus weight (317) and rats immunized with sheep red blood cells showed decreased serotonin content in the ventral part of the anterior hypothalamus (317), suggesting that serotonin also has anti-inflammatory and immunosuppressive actions (318). Serotonin was found to inhibit oxidative burst of human phagocytes and myeloperoxidase activity in a dose-dependent fashion (319). Serotonin inhibited TNF and IL-12 production but increased that of IL-10, NO, and PGE2 and these actions seem to be mediated through PGE2 (320). Serotoninergic receptors (5-HTR) are expressed by several inflammatory cell types, including DCs. Serotonin increased IL-6 production, induced maturation of DCs that enabled them to secrete high amounts of IL-10, and favored the outcome of a Th2 immune response both *in vitro* and *in vivo* (321). Thus, serotonin is a potent regulator of immune response and has pro-inflammatory actions and has a modulatory influence on mast cells (322). On the other hand, as shown in Figure 9, we noted that serotonin has cytoprotective actions at least against STZ-induced toxicity to RIN (pancreatic β) cells *in vitro* and it is possible that it may have similar action in an *in vivo* situation too. The modulatory influence of serotonin on immune response and inflammation may depend on the context and dose of the serotonin produced at the target tissue.

## Serotonin Regulates Insulin Secretion and Enhances β Cell Proliferation

The gut is rich in ECs, which release serotonin in response to food in the lumen that enters the circulation leading to an increase in the level of free serotonin in the blood that activates its receptors. High-fat diet fed rats showed increased levels of serotonin compared to the control while serotonin reuptake transporter (SERT)-dependent uptake of serotonin was reduced due to the upregulation of 5-HT synthesis genes and decreased reuptake and increased numbers and/or serotonin content of EC cells in the ileum (323). By contrast, a significant decrease in the total number of EC cells per crypt, a reduction in the levels of serotonin with no change in SERT-dependent uptake of serotonin and a lack of change in SERT protein levels associated with no change in tryptophan hydroxylase 1 mRNA was reported to occur in high-fat diet fed rats compared to the control in rat colon (324). This suggests that high-fat diet (HFD) leads to decreased serotonin availability in colon in response to HFD. This diametrically opposite changes in the levels of serotonin in different parts of the gut in response to HFD is rather surprising and suggests that serotonin may have a role in obesity and type 2 DM. This is supported by the observation that 5-HT receptor is involved in glucose regulation. Several 5-HT-receptor agonists, including selective serotonin reuptake inhibitors, which increase serotonin levels in the synaptic cleft, induce hyperglycemia (325–329). It is noteworthy that serotonin is present specifically in the pancreatic β cells and is secreted along with insulin in response to increase in blood glucose (330–335). Serotonylation, wherein serotonin covalently binds to the GTPase, is known to regulate insulin secretion. Lack of transglutaminase, which is essential for Serotonylation, leads to glucose intolerance (336). Mice that lacked peripheral tryptophan hydroxylase (Tph1 2/2), which is essential for serotonin synthesis (Tph 1 ^−/−^), developed diabetes and showed impaired insulin secretion, abnormalities that were restored to normal by administration of 5-hydroxytryptophan, the precursor of serotonin, that increased intracellular serotonin, bypassing the rate-limiting step (335, 337). Thus, 5-HT (serotonin) regulates insulin secretion (338). In addition, serotonin stimulates β cell proliferation (157). These results suggest that serotonin is not only needed for insulin secretion but also for β cell expansion when the need arises.

Smell of food increases appetite by enhancing dopamine release. Serotonin released during the consumption of food inhibits dopamine release by activating 5-HT2C receptors on dopamine-producing cells, and thereby serotonin decreases appetite. There is evidence to the involvement of serotonin, noradrenaline, and dopamine pathways in obesity and glucose homeostasis (339). Studies indicated that serotonin (5-HT) receptor 2A (5-HT2A), and 2C (5-HT2C) have a role in the regulation of appetite and energy homeostasis (340). Transcripts of the serotonin receptor are present in the hypothalamus, including in the paraventricular nucleus, lesions of which can result in obesity (341). Similarly, drugs that block 5-HT_2C_ receptors can facilitate weight gain (342), especially those who have low number of receptors (340). Serotonin release in the ventromedial nucleus is at peak in the morning, when the motivation to eat is strongest (343). These results suggest (157, 323–342) that serotonin plays not only a critical role in the pathobiology of obesity and type 2 DM but also interacts with other hypothalamic neurotransmitters, such as dopamine to regulate food intake, appetite, satiety, and β cell function, and these neurotransmitters regulate immune response, inflammation, and appetite and food intake and, thus, participate in the pathobiology of obesity and type 2 DM as discussed briefly below and summarized elsewhere (313, 344).

### Neuropeptide Y

Both sympathetic ganglia and leukocytes expressed high amounts of NPY mRNA and peptide. Leukocyte NPY expression was found to be much less during acute allograft rejection, an indication that it (NPY) could have a role in immune response and inflammation (345). Granulocyte accumulation into carrageenan-induced air pouch, phagocytosis, and peroxide production by leukocytes were inhibited, whereas NO generation was increased by NPY (346), indicating that NPY has anti-inflammatory actions. All these actions of NPY are mediated by its Y1 receptor. There seems to be an age-dependent modulation of inflammatory process by NPY (347).

Studies suggested that NPY increases nNOS (neuronal nitric oxide synthase) and, thus, modulates oxidative stress and subsequent inflammation, suggesting the close interaction among NPY, NOS, and pro-inflammatory cytokine TNF-α (348). It is noteworthy that gastrin-releasing peptide (GRP), NPY, somatostatin, and vasoactive intestinal peptide (VIP) stimulated the production of IL-1β in old subjects, and NPY, somatostatin, and VIP in young ones, whereas GRP, NPY, and VIP enhanced IL-6 production in young and old people. The TNF-α production was stimulated by NPY and somatostatin in young subjects and by NPY, somatostatin and VIP in old subjects, GRP decreased production of TNF-α in young persons. GRP in old subjects and VIP in young and old subjects stimulated LPS-induced IL-6 production by whole blood cells, whereas GRP and VIP suppressed LPS-induced TNF-α production in the young (348). These results suggest that neuropeptides have immunomodulatory actions in addition to their role in the control of appetite and food intake (313).

### Ghrelin

Human T lymphocytes and monocytes express ghrelin, an orexigenic peptide produced by the gut and acts on the growth hormone secretagogue receptor (GHS-R), was found to inhibit expression of IL-1β, IL-6, and TNF-α (313). Ghrelin suppressed and leptin enhanced human T lymphocyte GHS-R expression and, thus, have opposite actions on inflammation. Ghrelin has anti-inflammatory while leptin has pro-inflammatory action, suggesting a close relationship between energy metabolism and immune system (349, 350).

Tumor necrosis factor-α, a pro-inflammatory molecule, is known to play a role in depression, schizophrenia, and other psychiatric disorders and can cause anorexia in patients with cancer and tuberculosis. By contrast, ghrelin controls eating behavior by regulating the expression of orexigenic peptides in the hypothalamus and increases food intake and bodyweight. In general, weight loss increases ghrelin levels. In addition to its anti-inflammatory actions, ghrelin has antidepressant and anxiolytic actions (351–353). It is noteworthy that ghrelin downregulates pro-inflammatory cytokines in sepsis through activation of the vagus nerve (353), indicating that acetylcholine (Ach), the principal neurotransmitter of vagus, has potent anti-inflammatory actions (354, 355). This implies that TNF-α and ghrelin have opposite effects in the hypothalamic regulation of eating behavior, modulation of the immune response and the state of mental health and Ach suppresses TNF-α production. Similarly, hypothalamic monoamines serotonin, dopamine, and ACh and NPY, BDNF and melanocortins, not only modulate eating behavior but also participate in the regulation of immune response and inflammation (313, 350).

### Melanocortin

The proopiomelanocortin (*POMC*) gene transcribed by the anterior pituitary, hypothalamic arcuate nucleus (ARC) neurons, and cells of the dermis and the lymphoid system leading to the formation of *N*-terminal peptide, joining peptide, ACTH and lipotropin. Hypothalamus produces α-, β-, and γ-MSH but not ACTH. Melanocortin peptides act through five G-protein-coupled seven transmembrane domain receptors (melanocortin receptor type 1 [MC1R–MC5R]) and, thus, control food intake and energy balance by its (especially α-MSH) anorectic actions. By contrast, NPY and AgRP are orexigenic peptides and AgRP antagonizes the actions of MC3R and MC4R. NPY/AgRP neurons increase food intake and decrease energy expenditure, while POMC neurons have opposite actions. Leptin receptor is expressed on the arcuate neurons and during fasting both leptin levels and POMC mRNA are decreased with a concomitant increase in AgRP mRNA in the hypothalamus. POMC and AgRP have significant projections arising from ARC to several hypothalamic regions, including the lateral hypothalamus and the PVN. Lateral hypothalamus contains the melanin-concentrating hormone, whereas PVN contain thyrotropin-releasing hormone (TRH) through which melanocortin peptides exert their effects. Melanocortins have anti-inflammatory actions by their direct effects on the cells of the immune system and by affecting the function of resident non-immune cells and suppressing NF-kB activation, expression of adhesion molecules, and chemokine receptors, and by inhibiting the production of pro-inflammatory cytokines and other mediators (313, 356, 357). Thus, there is a very close, intricate and positive and negative feedback regulatory control among various hypothalamic neurotransmitters and neuropeptides that ultimately control appetite, satiety, food intake, inflammation, immune response, and energy homeostasis (313, 358).

### Acetylcholine

Several studies showed that ACh, the principal vagal neurotransmitter, has potent anti-inflammatory actions by its action on the α7 subunit-containing nicotinic ACh receptor (α7nAChR) (359–361). In addition, ACh has a modulatory influence on the production and actions of several hypothalamic monoamines and peptides, such as serotonin, dopamine, NPY, BDNF, and melanocortins. Thus, ACh is an important regulator of energy homeostasis and inflammation.

### Adrenaline and Noradrenaline

Subjects with stress hyperglycemia and type 2 DM are known to have enhanced levels of noradrenaline and adrenaline and diminished levels of serotonin and its metabolites in the brain, and augmented production and release of catecholamines from their circulating phagocytes. Furthermore, sympathetic activation is known to be associated with type 2 DM and metabolic syndrome and enhanced risk of cardiovascular disease. Type 2 DM is associated with an increase in the markers of inflammation and associated with cardiac sympathetic predominance (319, 362). Adrenaline and noradrenaline are pro-inflammatory in nature that supports the existence of low-grade systemic inflammation in metabolic syndrome that could be attributed to enhanced sympathetic activity. Since under normal physiological conditions, a balance is maintained between sympathetic and parasympathetic tones these results imply that type 2 DM and metabolic syndrome will be associated with decreased levels of plasma or tissue and leukocyte ACh levels, which have anti-inflammatory action with a concomitant increase in the production and release of catecholamines that ultimately results in sympathetic over-activity. It is interesting that Ach enhances eNO generation (363); PUFAs enhance Ach levels in the brain (364), augment insulin action (365, 366), and stimulate eNO generation (367, 368); insulin restores and protects Ach in intestinal colonic interstitial cells of Cajal in type 2 DM (369) and both insulin and Ach have anti-inflammatory actions (224, 225, 359–361), which suggests a close interaction(s) among neurotransmitter Ach, eNOS, pancreatic β cell insulin, and cell membrane lipid component PUFAs (313, 358). Thus, neurotransmitters (such as Ach and serotonin), chemical mediators (such as IL-6 and TNF-α), insulin and gases (such as NO) link peripheral (plasma glucose levels) events to central centers (hypothalamus) by a finely tuned yet complex set of molecules and mechanisms (see Figures 10 and 11).

## GLP-1 Modulates Inflammation

Incretins: GLP-1 and gastric inhibitory peptide (GIP), secreted by the intestinal L-cells, enhance insulin release after eating much before blood glucose levels are elevated. The enzyme dipeptidyl peptidase (DPP)-4 inactivates both GLP-1 and GIP. GLP-1 enhances insulin secretion in a glucose-dependent manner decreases glucagon secretion, and can increase β-cell mass. GLP-1 also suppresses acid secretion and gastric emptying and, thus, ultimately decreases food intake and enhances insulin sensitivity. Furthermore, GLP-1 is an immunomodulator and anti-inflammatory molecule.

Both astrocytes and microglia show GLP-1 binding and GLP-1 receptor mRNA expression and GLP-1 treatment produces morphological changes in microglia. GLP-1 suppressed LPS-induced IL-1β mRNA expression, and augmented cAMP concentration and cAMP response element-binding protein phosphorylation in astrocytes, implying that GLP-1 modulates inflammation (370).

Glucagon-like peptide-1 increased β-cell proliferation threefold in cytokine-treated cultures and restored to normal cytokine-reduced islet cell ERK1/2 activation and β-cell proliferation (371). These results suggest that GLP-1 has anti-inflammatory actions and can enhance β cell proliferation and, thus, preserves insulin-secreting ability of β-cells. DPP-4 inhibitor, sitagliptin, prolongs islet graft survival by inhibiting migration of splenic CD4+ T cells (372, 373).

### Leptin

As already discussed above, leptin has pro-inflammatory actions. Leptin stimulates peripheral blood mononuclear cells and increases their IL-6 and TNF-α production (374). Leptin triggers apoptosis of hypothalamic neurons and reduces synaptic inputs in the ARC and lateral hypothalamus by activating inflammatory pathways (375–377). Based on these pieces of evidence, it is proposed that high-fat diet-induced increase in leptin aggravates inflammation that may result in apoptosis of hypothalamic nuclei leading to the onset of type 2 DM.

### Cholecystokinin (CCK)

The peptide hormone CCK is produced by the gut, brain, and pancreatic β-cells that has actions on digestion, satiety, and insulin secretion. Deletion of CCK reduces β-cell mass expansion and increases apoptosis, suggesting that CCK has cytoprotective actions. Transgenic mouse that expresses CCK in the β-cell in the lean state showed increased β-cell area even in old age, resisted STZ-induced diabetes and had reduced β-cell apoptosis. CCK overexpression protected β cells from cytokine-induced apoptosis (378). These results imply that gut peptides such as CCK regulate β cell mass and CCK receptor agonists may prevent obesity and diabetes. Furthermore, GLP-1 that is secreted by islet α cells also protects β-cells from apoptosis via cAMP-mediated mechanisms. GLP-1 stimulates β-cell CCK production and secretion via cAMP-modulated transcription factor and cAMP response element-binding protein (CREB) that are needed for CCK expression. It is interesting that CCK regulation by cAMP does not require glucose or insulin. β-cell cytoprotective action of GLP-1 against cytokine-induced apoptosis seems to be partially dependent on CCK receptor signaling (379). Thus, GLP-1 secreted by islet α cells stimulates CCK synthesis and secretion in a paracrine manner via cAMP and CREB, whereas both GLP-1 and CCK protect β-cells from apoptosis.

Dietary fat stimulates CCK receptors that suppress inflammation by stimulating the efferent vagus nerve and nicotinic receptors to inhibit IL-6 and TNF-α production and decreases bacterial translocation due to increased permeability of the gut mucosa. This anti-inflammatory action of CCK needs an intact vagus nerve, suggesting the existence of a neuroimmunologic pathway regulated by nutrition (380, 381). Thus, CCK is needed to prevent inflammation induced by high-fat diet.

### Roux-en-Y-gastric bypass (RYGB) and Brain and Gut

In this context, it is noteworthy that fasting glucose, insulin, ghrelin, and PYY were significantly decreased and free fatty acids (FFAs) was elevated postoperatively in obese non-diabetic patients after RYGB surgery. Insulin sensitivity increased following surgery. In these subjects, postprandially an increase in C-peptide, GLP-1, GLP-2, PYY, CCK, and glucagon (in response to the mixed meal) occurred, whereas total and active ghrelin, leptin, and gastrin decreased with no change for GIP, amylin, pancreatic polypeptide, and somatostatin. Thus, after RYGB, an increase in insulin secretion and insulin sensitivity occurred and intestinal hormones changed in the direction of reducing hunger (382). By contrast, when similar study was performed in those with type 2 DM and age- and BMI-matched controls who underwent RYGB, mucosal biopsies taken during surgery and enteroscopy done after 10 months after surgery showed that the density of cells that secrete GLP-1, CCK, and GIP increased after RYGB, which explains amelioration of diabetes and increase in insulin sensitivity after weight loss surgery (since GLP-1, CCK, and GIP have anti-inflammatory and insulin-sensitizing actions).

Previously, we showed in an animal model of obesity that underwent RYGB surgery α-MSH in arcuate, parvocellular parts of paraventricular nucleus (pPVN) and magnocellular parts of PVN (mPVN) increased compared with obese controls. 5-HT-1B-receptor in pPVN and 5-HT-1B-receptor in mPVN increased in RYGB compared with obese controls. These results suggest that RYGB induced weight loss could be due to hypothalamic downregulation of NPY and upregulation of α-MSH and serotonin (383). In obese patients with type 2 DM, RYGB not only normalizes glycemic control but also leads to food reward-related brain activation patterns that are different from those of obese patients with less-well-controlled type 2 DM and without bariatric surgery (384). These results indicate that some very specific changes occur in hypothalamus and other areas of brain about food reward processing especially in hypothalamic neurotransmitters and peptides and gut hormones implying a close cross talk between gut and brain (385, 386). It has not been shown but likely that RYGB may restore the synthesis and action of BDNF, and EFA metabolism, and enhance the formation of AA, EPA, and DHA and their anti-inflammatory metabolites: LXs, resolvins, protectins, and maresins to normal.

These pieces of evidence suggest that hypothalamic monoaminergic and peptide molecules and gut peptides regulate appetite, satiety and food intake, and immune response and inflammation. Thus, obesity, insulin resistance, type 2 DM, and metabolic syndrome are closely related to inflammation (313, 358).

## Hypothalamic Inflammation Occurs in Obesity and Type 2 DM

**Figure 12:**
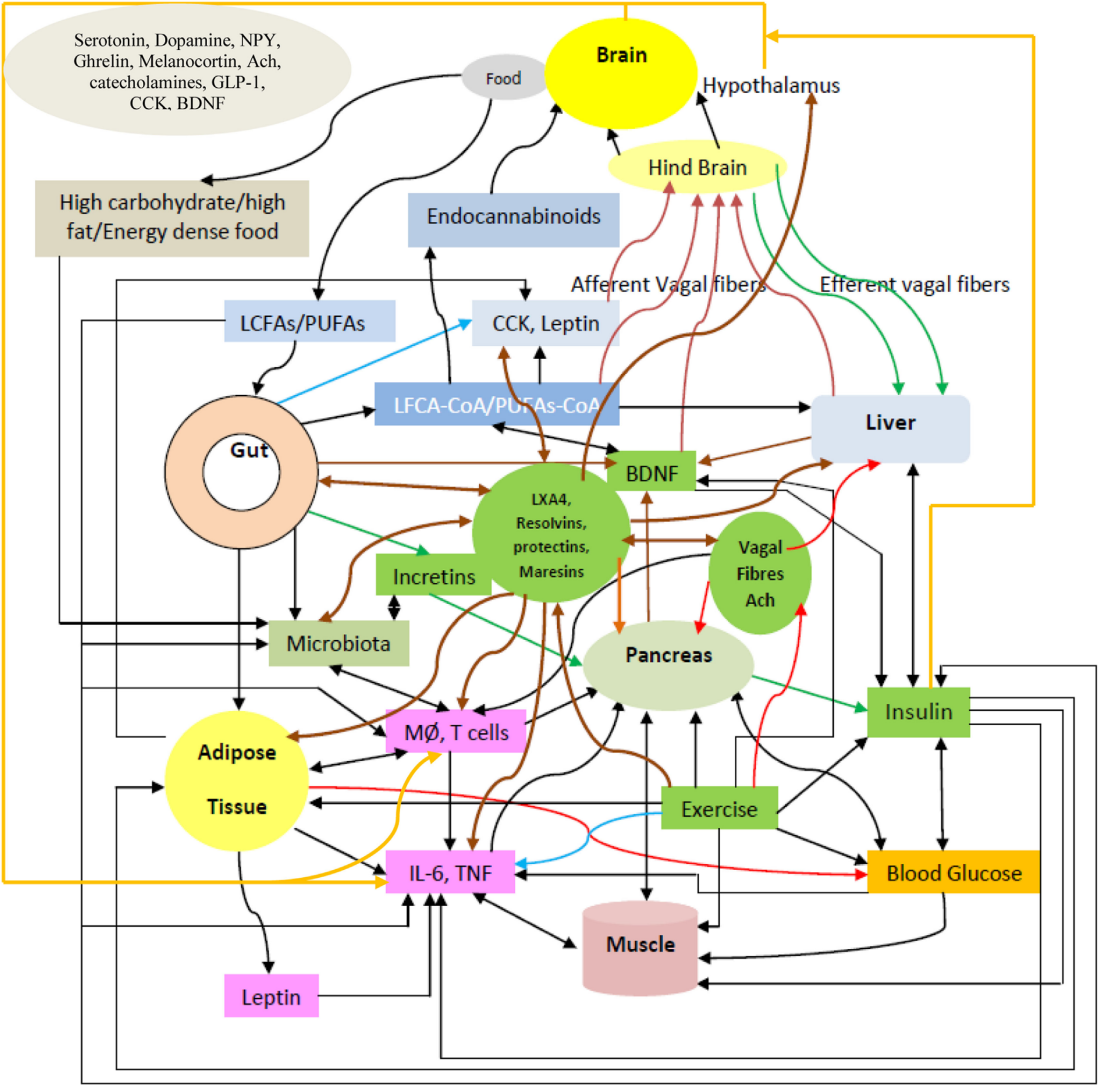
Scheme showing relationship among diet, gut microbiota, vagus, exercise, polyunsaturated fatty acid (PUFAs), lipoxins (LXs), resolvins, protectins, and maresins, and blood glucose, insulin, and tissues/organs concerned with glucose homeostasis: pancreas, muscle, liver, adipose tissue, and brain. High calorie diet induces low-grade systemic inflammation, obesity, and insulin resistance. PUFAs decrease insulin resistance, suppress secretion of pro-inflammatory cytokines, and lead to the formation of (a) LCFAs-CoA; (b) enhance gut cholecystokinin (CCK) secretion; and (c) augment endocannabinoids formation that act via afferent vagal fibers on hypothalamus to induce satiety and decrease appetite. PUFAs lead to increase in the formation of LXs, resolvins, protectins, and maresins that reduce insulin resistance, protect β cells from toxins, inhibit IL-6 and tumor necrosis factor-α (TNF-α) production, augment brain-derived neurotrophic factor (BDNF) production and action, interact with incretins, CCK, and acetylcholine, and influence gut microbiota, and may act on hypothalamic neurons and modulate insulin response of hypothalamic neurons and enhance response of peripheral tissues to insulin (reduce insulin resistance) and, thus, ameliorate type 2 DM. PUFAs enhance the growth of *Bacteroidetes* and inhibit *Firmicutes* and, thus, reduce obesity. PUFAs may augment the production and secretion of gut incretins that, in turn, augment insulin secretion. PUFAs enhance BDNF production, which inhibits appetite and decrease obesity. Liver and pancreas talk with each other through vagal fibers. Exercise reduces insulin resistance and obesity by (i) suppressing production of pro-inflammatory cytokines; (ii) increasing BDNF production in the brain and enhanced levels in the plasma; (iii) enhancing the production of lipoxin A4 (and possibly that of resolvins, protectins, and maresins) from muscle and gut; (iv) upregulating glucose utilization; (v) increasing vagal tone and thus, is (vi) anti-inflammatory in nature. Adipose tissue of obese subjects is infiltrated by macrophages and lymphocytes that secrete high amounts of IL-6 and TNF-α that cause low-grade systemic inflammation resulting in insulin resistance. Leptin has pro-inflammatory actions. *Bacteroidetes*, the predominant bacteria in the gut of the lean subjects, whereas *Firmicutes* are dominant in the gut of obese. *Firmicutes* breakdown polysaccharides and, thus, provide higher amounts of energy source that enhances the probability of development of obesity. *Firmicutes* stimulate gut associated lymphocytes and macrophages and augment production of pro-inflammatory cytokines. Insulin has anti-inflammatory actions and, hence, is likely that hyperinsulinemia seen in obesity and type 2 DM could be a compensatory phenomenon in order to suppress low-grade systemic inflammation seen in them. Insulin enhances activity of desaturases that leads to increased formation of arachidonic acid, eicosapentaneoic acid and docosahexaenoic acid, the precursors of LXs, resolvins, protectins, and maresins. Though expression and genotype (including single nucleotide polymorphism) of UCPs, FOXC2, adiponectin, FTO, MC4R, and other related genes are closely associated with obesity, their expression and function is modulated by diet, exercise, and other life-style-related factors. Thus, a close interaction(s) exists among genes, gut, diet, microbiota, and exercise that is not only complex but also interesting in the pathogenesis of obesity and type 2 DM. It may be noted here that PUFAs and acetylcholine and exercise can influence production and action of various neurotransmitters, such as serotonin, dopamine, leptin, ghrelin, GABA, and α-MSH and, thus, muscle, gut, food, and brain interact with each other and determine development/amelioration/prevention of obesity and type 2 DM.

High-fat diet has been shown to induce hypothalamic inflammation due to an HFD-induced increase in TNF-α that produces dysfunction of VMH neurons, leptin resistance, and defective regulation of energy homeostasis. Intracerebroventricular injections with antibodies against TLR-4 or TNF-α led to reversal of these abnormalities and improved insulin signaling in the liver and restored liver glucose production to normal. Vagotomy abrogated these beneficial effects. These results emphasize that hypothalamic inflammation that is seen in obesity and type 2 DM (387–389) is dependent on parasympathetic signals of the vagus nerve (359–361, 389). In fact, in a recent study, we observed that even in STZ-induced type 2 DM Wistar rats hypothalamic neuronal damage can be seen (see Figure 12).

On the other hand, PUFAs are neuroprotective by decreasing TNF-α production (390, 391), though some studies did not support this contention (392, 393). This discrepancy in the results is due to changes in the local production of various metabolites of PUFAs, such as prostaglandin E2 (PGE2), PGI2 and LXs, resolvins, protectins, and maresins (394). For instance, it is known that PGE2, formed from AA, suppresses IL-6 and TNF-α production yet it is pro-inflammatory in nature, whereas indomethacin, an anti-inflammatory compound, caused an increase in the TNF-α production by macrophages from experimental mice on high n-3 diet (394). On the other hand, LXs, resolvins, and protectins formed from AA, EPA, and DHA not only suppress IL-6 and TNF-α production but also have anti-inflammatory and wound healing actions (93, 98, 394–396). These paradoxical results suggest that the final outcome of AA, EPA, and DHA supplementation depends on the balance between pro- and anti-inflammatory molecules formed from them. Thus, the activities of COX and 5-, 12-, and 15-lipoxygnease enzymes and their expression in the target tissues determine what action is derived from the supplementation of various PUFAs. This implies that concentrations of TNF-α increase/decrease depends on the local levels of PUFAs and their metabolites. But, in general, decreased concentrations of PUFAs may enhance TNF-α production and this may produce neuronal damage leading to the onset of hypothalamic dysfunction and obesity and type 2 DM (313, 358).

The brain is rich in PUFAs (DHA > AA > EPA), which constitute as much as 30–50% of the total fatty acids in the brain. Hence, in the presence of inadequate concentrations of PUFAs especially, during the critical period of brain growth from the third trimester of pregnancy to two-year post-term, TNF-α concentration are likely to be high. This increase in TNF-α may damage VMH neurons and lead to the development of type 2 DM in adult life, indicating a significant role for TNF-α in the pathogenesis of type 2 DM. Thus, TNF-α has two important actions: (1) induction of peripheral insulin resistance and (2) induces apoptosis or interferes with the action of VMH and other hypothalamic neurons (313, 358).

It is interesting to note that insulin has anti-inflammatory actions and suppresses TNF-α production (253–258, 397); exercise enhances endogenous production of BDNF and LXA4 that possess antidiabetic actions; insulin receptors are present in the hypothalamus; insulin and BDNF and several hypothalamic peptides interact with each other; and BDNF and LXA4 enhance each other’s production. These positive and negative feedback regulations and interaction(s) among cytokines, insulin, hypothalamic peptides, BDNF, and PUFAs and their metabolites highlights the complexity of pathobiology of obesity and type 2 DM and the role of various tissues (hypothalamus, adipose tissue, muscle, liver, and immune system) in their pathobiology. This may also explain as to why exercise is such a potent regulator of obesity and type 2 DM since, regular and adequate exercise enhances production of BDNF and lipoxinA4 (101, 104, 263, 358, 389), two potent anti-obesity and antidiabetic endogenous molecules and regulators of inflammation. These actions seem to be independent of the energy expenditure property of exercise.

## Insulin and Insulin Receptors in the Brain and Type 2 DM

Insulin regulates food intake, neuronal growth, and differentiation and synaptic plasticity in the central nervous system by its ability to modulate release and action of various neurotransmitters. All features of type 2 DM, such as obesity, insulin resistance, and hyperinsulinemia, have been shown to occur in mice with neuron-specific disruption of the insulin-receptor gene (NIRKO) without any disturbance in brain development (398). These pieces of evidence suggest that in instances where there is a decrease in insulin receptor number and/or defects in insulin receptor function in the brain may lead to the development of type 2 DM. Inhibition of food intake can be induced by intraventricular injection of insulin by its action on the hypothalamic NPY network.

It is known that insulin augments the activity of Δ^6^ and Δ^5^ desaturases (see Figure 4) and, thus, enhances the formation of AA, EPA and DHA, the precursors of LXs, resolvins, protectins, and maresins. PUFAs enhance insulin action by increasing the number of insulin receptors by increasing cell membrane fluidity. In addition, both insulin and PUFAs enhance eNO formation, which could carry messages (probably via RBCs that carry NO) from VMH neurons to the pancreatic β-cells and, thus, regulate their insulin secretion. This indicates that presence of appropriate amounts of insulin and insulin receptors in the brain is essential to regulate appetite, obesity (BMI), maintain normoglycemia, and suppress inappropriate inflammation (313, 358, 398).

Thus, factors that regulate insulin action in the brain have a significant role in the control of type 2 DM that is supported by the observation that hypothalamus is rich in insulin receptors. Hence, development of drugs that bind to brain insulin receptors may decrease appetite, and reduce obesity and plasma glucose levels. It was reported that infusion of oleic acid (18:1 n-9) into the third ventricle decreases plasma insulin and glucose levels (399) by augmenting hepatic insulin action by activating K_ATP_ channels in the hypothalamus and suppressing hypothalamic NPY expression, the pieces of evidence that imply that PUFAs control food intake by their action on hypothalamus.

### BDNF in Obesity and Type 2 DM

There is considerable evidence to suggest that BDNF that is present in many tissues acts on hypothalamus, gut, and pancreatic β cells to regulate food intake and energy homeostasis (400–403). Systemic and intracerebroventricular administration of BDNF lowered blood glucose, decreased body weight, reduced hepatomegaly, and liver glycogen content in experimental animals with obesity and diabetes (403). Even BDNF administration once or twice per week is sufficient to lower blood glucose concentrations and hemoglobin A_1c_ (HbA_1c_) (404). BDNF regulates energy expenditure by activating sympathetic nervous system and its intracerebroventricular administration lowered blood glucose levels, enhanced insulin content in the pancreas, increased uncoupling protein-1 mRNA expression and augmented thermogenesis in db/db mice, events that can nullify or reverse DM and metabolic syndrome (404). These results coupled with the observation that serum BDNF levels are lower in type 2 DM (405) and that hyperglycemia, but not insulin, inhibited BDNF output from brain lends support to the argument that BDNF has a significant role in type 2 DM. Reports that plasma BDNF levels could be higher in some with obesity and DM can be attributed to the presence of resistance to the actions of BDNF or due to different methods employed in these studies. BDNF suppresses appetite and is expressed in VMH. Stress hormone corticosterone can suppress the expression of BDNF that may lead to atrophy of the hippocampus, which could be attributed to the absence of neurotrophic actions of BDNF (406, 407). This may explain the involvement of BDNF not only in obesity and type 2 DM but also its role in depression and Alzheimer’s disease (404, 405). In a recent study, we noted that BDNF enhances the synthesis and secretion of LXA4, a potent anti-inflammatory molecule. Thus, it is likely that when BDNF levels are low, it could lead to decreased production of LXA that, in turn, initiates and augments local inflammatory circuit. We also noted that LXA4 augments BDNF synthesis suggesting that cellular content of AA, the precursor of LXA4, has a regulatory role in the control of inflammation partly, by regulating BDNF and LXA4 concentrations. Hence, cellular content of AA and other PUFAs and the activity of desaturases (Δ^6^ and Δ^5^) and COX and 5-, 12-, and 15-lipoxygeanses that regulate the formation of LXs, resolvins, protectins, and maresins; and other eicosanoids that influence inflammation are likely to play a critical role in the pathobiology of obesity and type 2 DM (101, 104, 263, 358, 389, 394).

### Insulin, Melanocortin, and BDNF

In addition to its action on glucose metabolism and fatty acid synthesis following its binding to its receptor and translocation of Glut-4 transporter, insulin release itself is stimulated by food intake, ACh, and CCK, while norepinephrine (noradrenaline) inhibits its action that is responsible for stress hyperglycemia.

Insulin acts as an adiposity signal by acting on the ARC of hypothalamus (408). Insulin enhances POMC synthesis that, in turn, acts on melanocortin receptors MC3R and MC4R of hypothalamic nuclei (409). These melanocortin receptors regulate energy balance. BDNF is expressed by VMH neurons and both nutrition and MC4R regulate its (BDNF) expression. For instance, absence of MC4R reduces the expression of BDNF receptor TrkB to a quarter of its normal amount. These animals also show hyperphagia and weight gain on higher-fat diets. BDNF infusion into the brain decreased hyperphagia and weight gain when fed high-fat diet. These pieces of evidence suggest that MC4R signaling regulates BDNF expression and BDNF may function as one of the mediators of actions of MC4R, though this has been disputed (406, 410). It is noteworthy that LXA4 regulates BDNF synthesis and secretion. It is likely (but needs to be confirmed) that resolvins, protectins, and maresins may also have a regulatory role on BDNF synthesis. Since, synthesis of LXs, resolvins, protectins, and maresins depends on the release of PUFAs from the cell membrane phospholipids, cell membrane integrity, and their PUFA content is yet another critical factor in the regulation of synthesis and action of BDNF. Furthermore, cell membrane fluidity is an important factor that regulates the expression and affinity of receptors on cell membranes (294, 358). This, in turn, depends on the cell membrane content of various PUFAs: higher the PUFA content higher the fluidity of the membrane and higher the expression and affinity of the receptors to their respective peptides/hormones. These results imply that PUFAs and their metabolites, BDNF concentrations and expression and affinity of various hypothalamic neurotransmitters to their receptors are interconnected that ultimately regulates appetite, satiety, and hunger by influencing the actions of various peptides, including insulin, melanocortin, BDNF, and the onset or amelioration of type 2 DM (see Figure 12). PUFAs and their metabolites by their regulatory role in inflammation and immune response is yet another layer of complexity in the pathobiology of type 2 DM.

### Ghrelin, Leptin, and BDNF

Ghrelin, a gut hormone, is produced by the fundal epithelial cells of the stomach, placenta, kidney, pituitary, and hypothalamus. Ghrelin increases food intake and it enhances growth hormone secretion. Ghrelin acts on ARC of hypothalamus (411). Glucose utilization rate of white and brown adipose tissue is increased by ghrelin administration that can lead to an increase in body weight (412). Plasma glucose, insulin, ACh leptin, BDNF, and various neurotransmitters and peptides are some of the factors that have a regulatory role in its secretion and action (413–415).

Leptin, which is produced by several tissues including white adipose tissue, stomach, mammary gland, placenta, and skeletal muscle, has actions such as insulin and regulates appetite and obesity (416). Leptin acts on the hypothalamus to suppress NPY, increase POMC and corticotrophin-releasing hormone and TRH, and reduce MCH and orexins. In addition, leptin and BDNF interact with each other suggesting that BDNF may, at least, partially regulate leptin action (416–418).

### BDNF in Obesity and Type 2 DM

Thus, BDNF regulates energy homeostasis and plays a significant role in the pathobiology of type 2 DM by interacting with leptin, ghrelin, insulin, NPY, melanocortin, serotonin, dopamine, and other neuropeptides, neurotransmitters, PUFAs and LXA4, and gut hormones. BDNF can prevent exhaustion of the pancreatic β cells, especially in diabetic mice and, thus, able to restore the level of insulin-secreting granules in β cells (313, 358, 389, 419). BDNF administration can ameliorate diabetes in experimental animals (419). This suggests that methods designed to deliver BDNF in appropriate amounts may form a new approach to prevent or manage type 2 DM.

How these apparently disparate events and molecules can be integrated to the role of PUFAs in type 2 DM?

## Conclusion: PUFAs and Their Metabolites in DM

Based on the preceding discussion that PUFAs and their metabolites, hypothalamus and their peptides and neurotransmitters, BDNF, insulin receptors in the brain, gut peptides/hormones, cytokines, and gut microbiota play a role in obesity and DM, it is evident that all these factors/events are interrelated.

In a series of previous studies, we showed that oral feeding of oils rich in ω-3 EPA and DHA and ω-6 GLA and AA or pure FFAs (GLA, AA, EPA, and DHA) prevent apoptosis of insulin-secreting rat insulinoma (RIN5F) cells *in vitro* and alloxan-induced type 1 DM and STZ-induced type 1 and type 2 DM in experimental animals (15–18, 137, 420). This beneficial action of PUFAs against chemical-induced type 1 and type 2 DM is not abrogated by COX and LOX inhibitors, suggesting that fatty acids themselves are active and/or their anti-inflammatory LXs, resolvins, protectins, and maresins may have antidiabetic actions [see Figures 6–8 and Ref. (15–18, 137, 420)], while pro-inflammatory PGs, LTs, and TXs are ineffective (131, 132). Our recent studies showed that fish oil (a rich source of EPA and DHA) altered the growth of *Helicobacter, Clostridiales, Sphingomonadales, Firmicutes, Pseudomonas* species, and several other bacteria (421). These results are interesting because it is known that *Firmicutes* play a significant role in obesity, the precursor of type 2 DM (422, 423). Ghosh et al. (423) showed that n-6 PUFAs enriched the gut microbiota with *Enterobacteriaceae, Segmented Filamentous Bacteria, and Clostridia* species that are associated with or induce inflammation, whereas addition of n-3 PUFAs to a high n-6 PUFA diet reversed these inflammation-inducing microbial growths and enriched the gut with the beneficial microbes like *Lactobacillus* and *Bifidobacteria*. But, it is not known whether LXs, resolvins, protectins, and maresins have such influence on gut microbiota though they have been shown to alter growth of *E. coli*, partly by acting on neutrophils (424, 425). It is important to study whether PUFAs, LXs, resolvins, protectins, and maresins can influence Treg and Teff functions.

Arachidonic acid, EPA, and DHA are present in significant amounts in the brain. Plasma concentrations of PUFAs are low in patients with type 1 and type 2 DM (127, 244, 358). Expression of insulin and other receptors and the affinity of the respective proteins/hormones/peptides are altered depending on the cell membrane fluidity. Cell membranes that contain high amounts of PUFAs are more fluid and so the number of insulin receptors and their affinity to insulin will be higher that would ultimately reduce insulin resistance (294, 358, 426–429). These results imply that presence of adequate amounts of PUFAs enhance the action of insulin and BDNF on their target cells. In view of this, it is proposed that a combination of PUFAs and BDNF may prevent DM.

**Figure 13:**
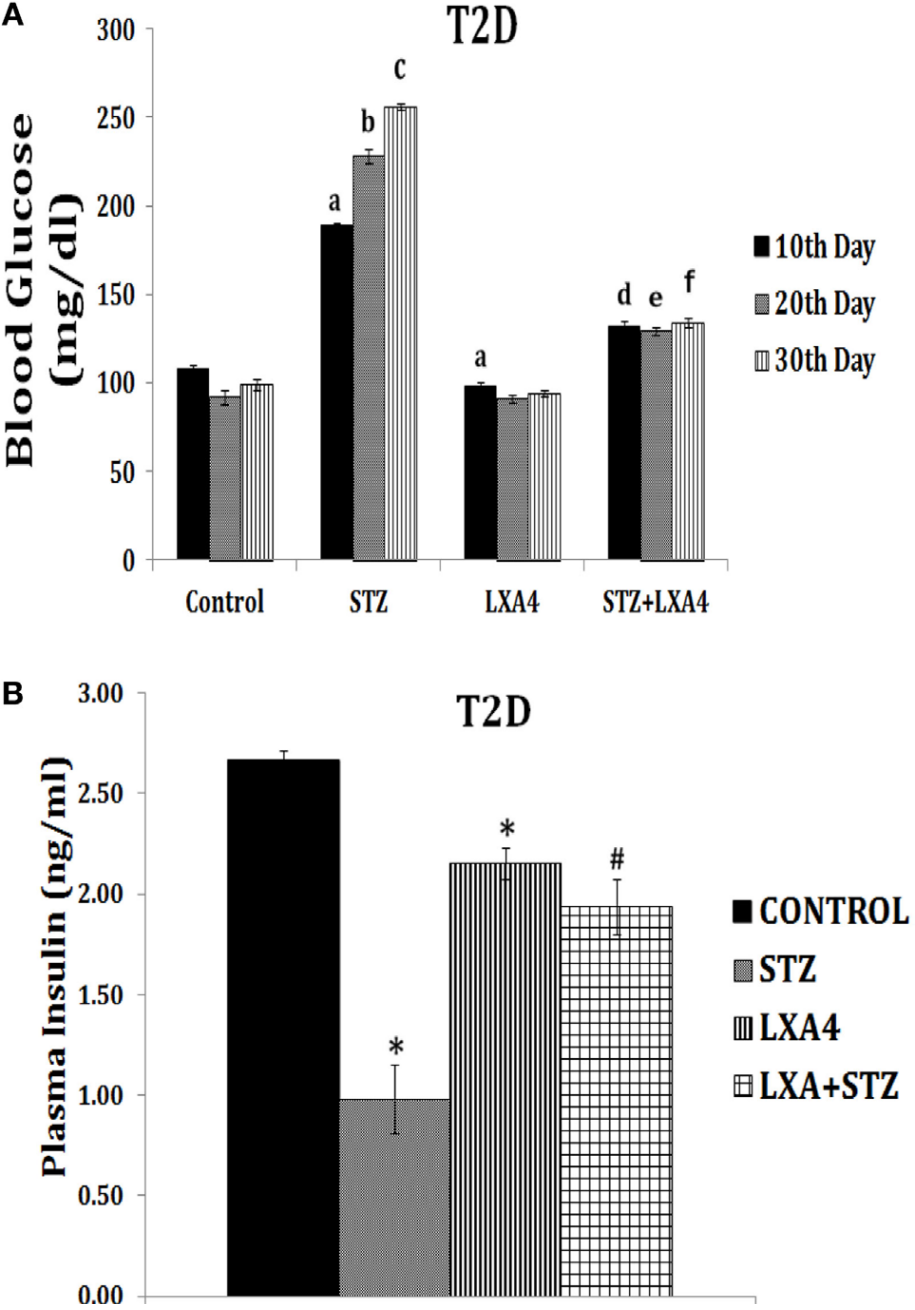
Effect of lipoxin A4 (LXA4) against streptozotocin (STZ)-induced type 2 DM in Wistar rats. *Protocol of the study*: after 7 days of acclimatization, type 2 DM was induced by intraperitoneal administration of STZ 175 mg/kg of body weight, which is considered as day 1 of the study. LXA4 60 ng/animal was given intraperitoneally on day 1 (the day STZ was administered) and daily for 5 days [these data are taken from Ref. (420)]. These studies were approved by Institutional Animal Ethics committee. **(A)** *Plasma glucose levels*: plasma glucose was estimated once in 10 days till day 30, the day study was concluded. All values are expressed as mean ± SEM. ^a^*P* ≤ 0.05 compared to control values of day 10. ^b^*P* ≤ 0.05 compared to control values of day 20. ^c^*P* ≤ 0.05 compared to control values of day 30. ^d^*P* ≤ 0.05 compared to STZ values of day 10.^e^*P* ≤ 0.05 compared to STZ values of day 20. ^f^*P* ≤ 0.05 compared to STZ values of day 30. *P ≤ 0.05 compared to untreated control. ^#^*P* ≤ 0.05 compared to STZ control. **(B)** *Plasma insulin levels measured on day 30 of the study*: all values are expressed as mean ± SEM. *P ≤ 0.05 compared to untreated control; ^#^*P* ≤ 0.05 compared to STZ.

**Figure 14:**
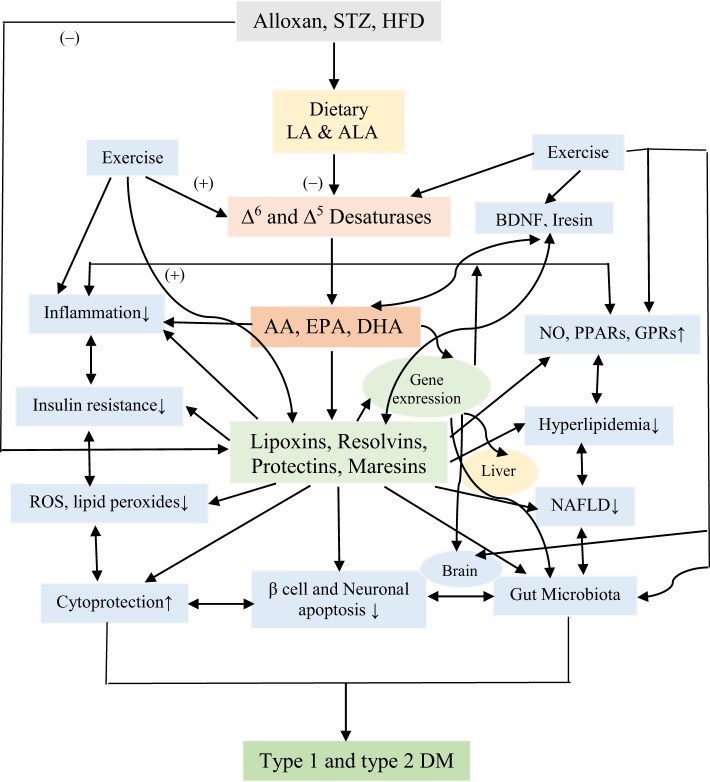
Scheme showing actions of polyunsaturated fatty acid and their anti-inflammatory products: lipoxins (LXs), resolvins, protectins, and maresins on various factors that have a role in the pathobiology of type 1 diabetes mellitus (type 1 DM) and type 2 diabetes mellitus (type 2 DM). (−) indicates inhibition of action or negative control. (+) indicates increase in action or synthesis or positive control. Exercise enhances the formation and action of brain-derived neurotrophic factor (BDNF), LXs, resolvins, protectins, and maresins and suppress inflammation and reduces insulin resistance. BDNF, LXs, resolvins, protectins and maresins suppress inflammation, reduce insulin resistance, and protect pancreatic β cells from the cytotoxic action various endogenous and exogenous cytotoxic molecules/agents. Alloxan, streptozotocin, and high-fat diet suppress the activities of desaturases and reduce the formation of LXs, resolvins, protectins, and maresins and their precursors and the formation and action of BDNF (for details see text).

It is interesting that AA and LXA4 prevented both alloxan-induced type 1 DM and type 1 and type 2 DM induced by STZ [see Figure 13, results with LXA4 only are shown (137, 420)]. LXA4 treatment decreased plasma TNF-α level. Expression of genes Pdx1 and IKB were increased while that of NF-kB was decreased in pancreatic tissue in LXA4-treated animals, suggesting that anti-inflammatory action is one of the mechanisms by which LXA4 can prevent type 2 DM. In addition, the expressions of lipocalin-2 and NF-kB were decreased, whereas that of IKB was enhanced in LXA4-treated animals in adipose tissue (420). It is worth noting that alloxan STZ and HFD inhibit the activity of both Δ^6^ and Δ^5^ desaturases and enhance concentrations of pro-inflammatory PGE2 and, thus, induce insulin resistance, the hallmark of obesity, type 2 DM and metabolic syndrome [unpublished data, see Figure 4, and Ref. (53, 101, 358, 366)]. It is likely that HFD increases pro-inflammatory PGE2, decrease the activities of desaturases resulting in a deficiency of AA, EPA, and DHA, the precursors of LXs, resolvins, protectins, and maresins, and a concomitant decrease in BDNF production (since LXA4 and PUFAs enhance the production of BDNF and so a deficiency of LXA4 and AA, EPA, and DHA results in decreased formation of BDNF) that will tilt the balance more in favor of pro-inflammatory status resulting in insulin resistance, dysfunction of pancreatic β cells, and finally development of type 2 DM and when destruction of β cells occurs leads to type 1 DM. Since PUFAs are also able to alter gut microbiota (421, 422), neurotransmitter release, and action [especially that of ACh (313, 350, 430, 431)], enhance BDNF synthesis and secretion [unpublished data (431)], and LXA4 enhances BDNF secretion and vice versa, modulate immune response and suppress IL-6 and TNF-α synthesis (92, 93, 98, 389–391, 394–396), gut hormone release (including that of GLP-1) (432–434), and finally may alter gene expression as well (277, 435–438). These and other pieces of evidence (439–441) as discussed above attest to the interesting possibility that PUFAs (especially AA) and their metabolites (especially LXA4) may play a significant role in the pathogenesis of both type 1 and type 2 DM (see Figure 14). Hence, it is worthwhile to explore novel methods of delivery of these molecules in the prevention and management of DM.

## Author Contributions

The author confirms being the sole contributor of this work and approved it for publication.

## Conflict of Interest Statement

The author declares that the research was conducted in the absence of any commercial or financial relationships that could be construed as a potential conflict of interest.
